# Non-degeneracy of coated laminates with a degenerate core

**DOI:** 10.1007/s00707-026-04710-9

**Published:** 2026-05-12

**Authors:** Jonas Lendvai, Matti Schneider

**Affiliations:** 1 Institute of Engineering Mathematics, University of Duisburg-Essen, Essen, Germany; 2 Fraunhofer Institute for Industrial Mathematics ITWM, Kaiserslautern, Germany; 3 Center for Nanointegration Duisburg-Essen (CENIDE), Duisburg, Germany

## Abstract

Coated laminates arise from sequential lamination, where a core phase is repeatedly embedded into a coating phase along varying lamination directions and volume fractions. For non-degenerate material parameters, explicit formulas for the effective conductivity and stiffness of such microstructures are well established. This work investigates coated laminates with a *degenerate core*, including insulating and perfectly conducting phases in thermal conductivity as well as porous and rigid phases in linear elasticity. Motivated by observations in deep material networks, we analyze under which conditions the effective tensors of a coated laminate become non-degenerate despite the degeneracy of the core. We develop an abstract operator-theoretic framework in a Hilbert space setting that encompasses both conductivity and elasticity and allows a unified treatment of vanishing and infinite material parameters. Within this framework, we establish well-posedness of the coating process and derive explicit criteria for non-degeneracy in terms of intersection properties of suitable subspaces. The abstract results are subsequently specialized to thermal conductivity and linear elasticity, where the derived criteria yield explicit conditions for the non-degeneracy.

## Introduction

### State of the art

Layered or laminated materials vary only along a single spatial direction and thus constitute one of the simplest non-trivial classes of composite microstructures. This layering direction is given by the lamination normal ${ n}$, a unit vector characterizing the orientation of the material interface. Despite this apparent simplicity, laminates show anisotropic effective behavior and admit explicit or semi-explicit homogenization formulas within the general framework of mathematical homogenization theory [1–3]. These formulas relate the macroscopic effective response to the underlying geometrical and material parameters under the assumption of scale separation, i.e., in the limit of vanishing microstructural length scale [4, 5]. For this reason, laminates played an important role in the early stages of homogenization theory and composite materials science. More generally, laminates serve as canonical examples where the relation between microstructure and macroscopic properties can be analyzed via explicit analytic formulas. This property makes them particularly useful as reference configurations for validation and benchmarking, since analytically solvable microstructures are rare in mechanics and related fields. In linear elasticity, the long-wavelength effective response of finely layered media whose properties vary only along a single spatial direction is classically described by the Backus average [6], which builds on earlier analyses of wave propagation in stratified elastic media [7] and yields closed-form expressions for the effective stiffness tensor and the local fields. Similar explicit formulas were derived for laminated piezoelectric composites and related coupled systems [8], and the same averaging framework extends to poro-elastic media, where transversely isotropic effective behavior arises from thin layers with isotropic material properties [9].

Beyond simple rank-one laminates, where two homogeneous phases are combined by a single lamination step, hierarchical or multiple-rank laminates arise by sequential lamination on widely separated length scales and along different directions. Here, the rank denotes the number of successive lamination steps, while a phase refers to a homogeneous constituent material characterized by its own material properties. Such hierarchical microstructures were already considered in early work by Maxwell [10], who derived formulas for the effective electrical conductivity of higher-rank laminates under specific structural assumptions. It was subsequently shown that appropriately designed multiple-rank laminates closely approach rigorous bounds on effective conductivity tensors of statistically isotropic polycrystals [11]. This insight forms a cornerstone of modern composite theory and is developed systematically in the comprehensive treatment by Milton [12, §9], where general lamination formulas are derived for thermal and electrical conductivity as well as for linear elasticity. In particular, laminates frequently appear as extremal microstructures in variational bounds such as the Hashin–Shtrikman bounds [13] and their anisotropic extensions [14, 15]. The explicit characterization of these bounds and their attainment by laminate constructions continues to inform modern surrogate modeling strategies, where admissible solution spaces are restricted a priori by enforcing Voigt- and Reuss-type bounds through spectral normalization techniques and related neural architectures  [16, 64]. Beyond the linear regime, high- and infinite-rank sequential laminates were employed to model the effective nonlinear behavior of composites, including power-law and transport problems, and were shown to converge toward effectively isotropic responses as the rank increases, provided the lamination directions are suitably distributed on the unit sphere [17–19]. More recently, laminated microstructures composed of elasto-plastic single-crystal phases were analyzed within gradient-enhanced crystal plasticity frameworks, allowing size-dependent and orientation-dependent effects to be captured analytically [20].

**Fig. 1:**
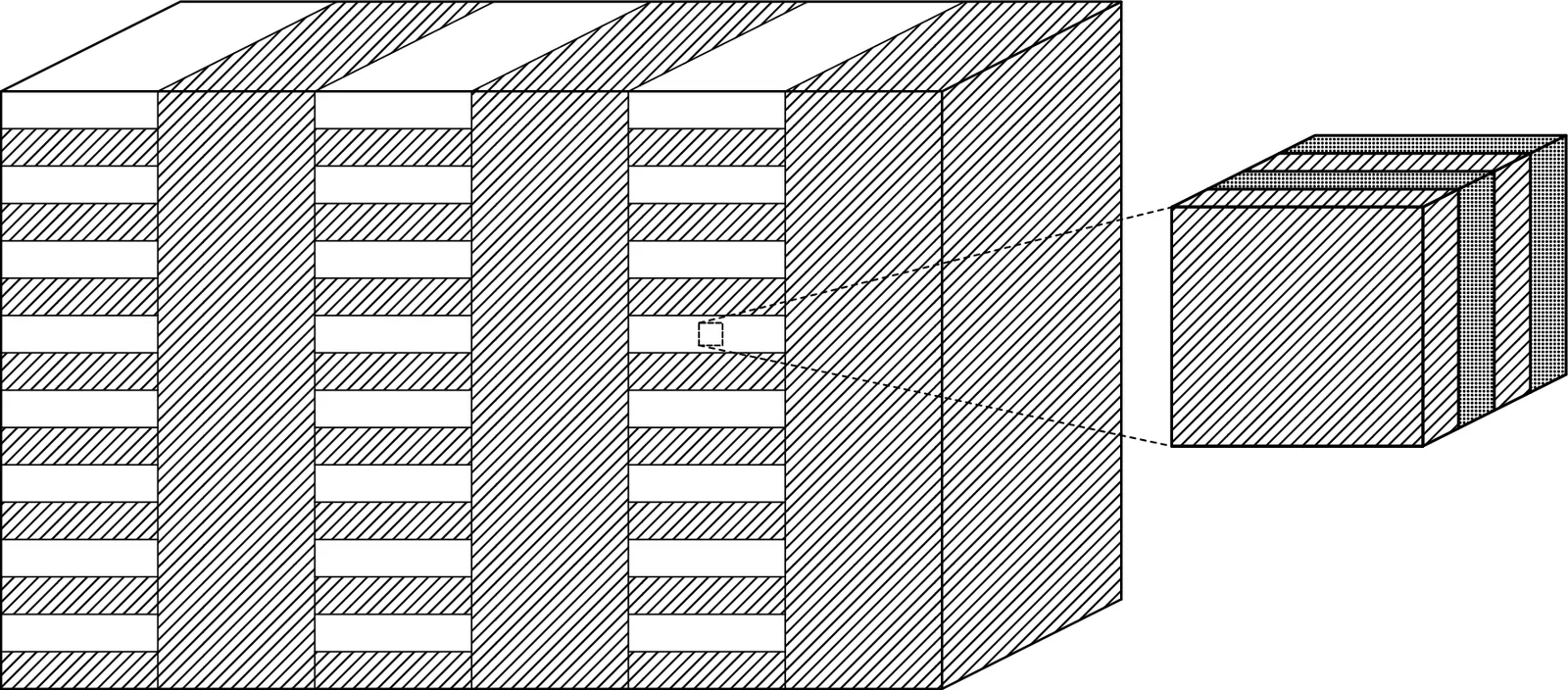
Rank-three coated laminate with mutually orthogonal layering directions. The core phase is depicted by a dotted pattern, while the coating phase is shown hatched. The white region corresponds to the laminated microstructure illustrated in the zoomed view. The separation of length scales between the successive layering steps is indicated by the different layer thicknesses

Among hierarchical constructions, a particularly important subclass is formed by *coated laminates*, also referred to as matrix laminates, which are obtained by sequentially laminating a given core phase into a surrounding matrix phase, such that at each step the previously formed laminate is embedded into an additional layer of the coating material along a new direction, as illustrated in Fig. 1. In the context of two-phase conductivity, explicit lamination formulas for simple and coated laminates were developed independently, providing closed-form representations of the effective response in terms of lamination directions and volume fractions [21, 22]. A systematic presentation of these constructions, including explicit formulas for the effective conductivity and elasticity tensors of coated laminates, is given by Milton [12, §9.8].

Although these constructions emerged in the context of classical analytical homogenization, the underlying laminate concepts gained renewed relevance in modern computational and data-driven multiscale modeling. In FFT-based computational homogenization [23, 24], explicit laminate formulas are exploited locally to represent interfaces and unresolved sub-voxel microstructures, leading to the so-called laminate composite voxels [25–28]. In a different direction, hierarchical laminates form the structural backbone of deep material networks (DMNs), which were originally introduced as differentiable trees of sequential laminates mapping the constitutive behavior of the individual phases to the effective composite response [29–31]. Recent review articles provide comprehensive overviews of the methodology and its developments [32–34]. For this construction, each node of the tree represents a two-phase laminate, while rotations transfer information between different lamination directions, yielding a hierarchical operator representation of the microstructure. The framework was subsequently placed on a rigorous micromechanical foundation by recasting the DMN in an operator learning setting and demonstrating that thermodynamic consistency and classical bounds such as the Voigt and Reuss estimates are inherited directly from the laminate building blocks [35]. Significant computational advances were achieved through a reduced laminate parameterization and a flattened online formulation, which—combined with sparse implementations and pruning—enabled large accelerations and fully coupled FE–DMN simulations [35, 36]. More recently, improvements of the offline training phase were proposed via a displacement-based laminate formulation [37], building on displacement-based laminate representations [26, 38, 39], which substantially reduce the computational cost during the networks’ training. The laminate-based architecture further proved flexible with respect to constitutive complexity, with extensions to cohesive zone models [40], damage and strain localization [41], fatigue damage [42], coupled plasticity–creep formulations [43], fully coupled thermomechanical models [44, 45], thermal conductivity [46] and crystal plasticity [47]. Recent developments further address extreme material contrast and degenerate phases, including applications to the viscosity of particle suspensions [48], underscoring the robustness of hierarchical laminate constructions even in singular parameter regimes.

### Contributions

Classically, laminate formulas are derived by two complementary approaches: either via interfacial jump balances, where stress and strain discontinuities across the interface are expressed in terms of the material tensors [49–52], or via the kinematic assumption of piecewise homogeneous stress and strain fields within each layer, which relies on scale separation between lamellae and macroscopic loading [38, 53, 54]. Both approaches implicitly rely on the positive definiteness and on the invertibility of the difference of the material tensors, and therefore do not directly extend to singular parameter regimes.

In such singular limits, fundamental questions about the validity and stability of the coating procedure arise. If material parameters vanish or become unbounded, the algebraic manipulations underlying classical laminate formulas are no longer justified. At the same time, such singular material parameters naturally occur in porous media, rigid inclusions, high-contrast composites and explicitly within laminate-based surrogate models such as DMNs. A rigorous analysis of the coating process is therefore mandatory to determine whether and under which conditions successive laminations may restore coercivity. We therefore address the following question:

**Figure:**
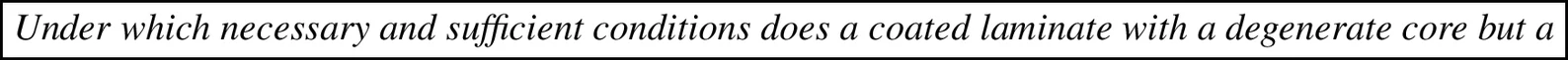

**Figure:**
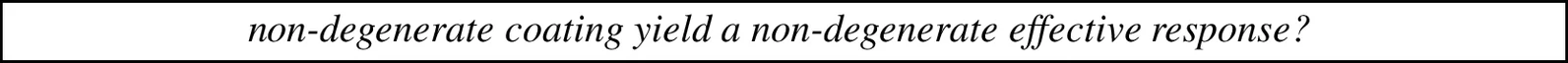

Here, non-degeneracy refers to the positive definiteness of the effective operator, i.e., coercivity of the corresponding constitutive mapping in conductivity or linear elasticity.

To illustrate the problem, consider thermal conductivity with an insulating core and a coating of finite isotropic conductivity. A simple rank-one laminate remains insulating in the lamination direction, whereas a second lamination in a non-collinear direction may restore finite conductivity. In linear elasticity, similar phenomena arise for porous or rigid cores. Numerical evidence originating from DMNs suggested that two laminations are insufficient to guarantee a non-degenerate effective stiffness, while three laminations with linearly independent normals empirically restore positive definiteness [55].

The present work provides a rigorous mathematical foundation for these observations. We develop an abstract formulation of the coating process in a Hilbert space setting, encompassing thermal conductivity and linear elasticity using vanishing and infinite material parameters. Within this framework, coated laminates are described in terms of positive self-adjoint operators, allowing a unified treatment of degeneracy.

The novelties of the developments include:

- Classical lamination formulas [12, §9.4] require the difference between the material tensors of coating and core to be invertible. We show that this assumption is not necessary.
- We provide a direct derivation of the classical laminate formula [12, §9.3] from the primal Lippmann–Schwinger equation [24, 56, 57] in elasticity, thereby establishing the correspondence between both formulations in a transparent manner. The derivation is presented in Appendix A.
- We establish well-posedness of the coating process in the presence of degeneracy and derive necessary and sufficient conditions for the resulting effective operator to be non-degenerate, providing an explicit characterization in terms of intersection properties of suitable closed linear subspaces.
- Instead of inferring symmetry and positivity from microstructural arguments, we provide a direct operator-theoretic derivation of these properties within the abstract framework.

The remainder of this work is organized as follows. In Sect. 2, we recall the classical effective formulas for simple and coated laminates in linear elasticity and thermal conductivity, providing the analytical background for the subsequent developments. Section 3 introduces the abstract Hilbert space framework for the coating process. After revisiting simple two-phase lamination, we formulate coated laminates in the operator-theoretic setting and derive necessary and sufficient conditions for non-degeneracy in the presence of a degenerate core. Section 4 returns to the concrete physical settings of elasticity and conductivity and discusses the implications of the abstract results for these model problems.

## The effective properties of laminates and their coated cousins

### Linear elasticity

Suppose that a laminated elastic material is given, i.e., a stiffness tensor field $\mathbb {C}$ depending on the microstructure position ${ x}$ that varies only along a fixed lamination direction ${ n}$, a fixed unit vector ${ n}\in \mathbb {R}^d$. Then, the effective stiffness $\mathbb {C}^{\text {eff}}$ of this material may be expressed in the following implicit form [12, §9]

2.1 $$\begin{aligned} \left[ (\mathbb {C}^{\text {eff}} - \mathbb {C}^0)^{-1} + \hat{\mathbb {\Gamma }}^0({ n})\right] ^{-1} = \left\langle {\left[ (\mathbb {C}- \mathbb {C}^0)^{-1} + \hat{\mathbb {\Gamma }}^0({ n})\right] ^{-1}} \right\rangle , \end{aligned}$$

involving an elastic reference material $\mathbb {C}^0$ that is either sufficiently stiff of sufficiently compliant [25], and where the angular brackets stand for a volume average in periodic homogenization and an ensemble average in stochastic homogenization. The fourth-order tensor $\hat{\mathbb {\Gamma }}^0({ n})$ acts on a stress $\boldsymbol{\tau }\in \text {Sym}(d)$ via

2.2 $$\begin{aligned} \hat{\mathbb {\Gamma }}^0({ n}):\boldsymbol{\tau }= { n}\otimes _s \left[ \hat{\boldsymbol{ A}}^0({ n})(\boldsymbol{\tau }\cdot { n})\right] , \end{aligned}$$

in terms of the acoustic tensor associated with the reference stiffness $\mathbb {C}^0$ in direction ${ n}$, namely the second-order operator $\hat{\boldsymbol{ A}}^0({ n})$ relating tractions on planes normal to the direction ${ n}$ to the corresponding displacements, implicitly defined by

2.3 $$\begin{aligned} \left[ \hat{\boldsymbol{ A}}^0({ n})\right] ^{-1} { u}= { n}\cdot \left[ \mathbb {C}^0 : ({ n}\otimes _s { u})\right] , \qquad { u}\in \mathbb {R}^d, \end{aligned}$$

where $\otimes _s$ denotes the symmetrized tensor product. In the special case where the reference stiffness is proportional to the identity on $\text {Sym}(d)$, i.e., $\mathbb {C}^0 = \lambda \,{{\,\mathrm{\text {Id}}\,}}$ with $\lambda \in \mathbb {R}$, it is convenient to separate the purely geometric contribution associated with the lamination direction from the effect of the reference material. To this end, the operator $\hat{\mathbb {\Gamma }}^0({ n})$ may be written as

2.4 $$\begin{aligned} \hat{\mathbb {\Gamma }}^0({ n}) = \hat{\mathbb {\Gamma }}({ n}) : \left[ \mathbb {C}^0\right] ^{-1}, \end{aligned}$$

where $\hat{\mathbb {\Gamma }}({ n})$ is a fourth-order projection operator on $\text {Sym}(d)$ depending only on the direction ${ n}$, which admits the explicit representation in index notation

2.5 $$\begin{aligned} \hat{\mathbb {\Gamma }}_{ijk\ell }({ n}) = \frac{1}{2}\left( n_i \delta _{jk} n_\ell + n_i \delta _{j\ell } n_k + n_j \delta _{ik} n_\ell + n_j \delta _{i\ell } n_k \right) - n_i n_j n_k n_\ell . \end{aligned}$$

The geometric structure of the tensor $\hat{\mathbb {\Gamma }}({ n})$ is closely related to the fourth-order interfacial operators [58], which provide an algebraic framework for decomposing stress and strain fields into components normal and tangential to material interfaces.

There are different ways to establish the validity of Eq. (2.1), see Milton [12, §9]. Actually, there is a correspondence between Lippmann–Schwinger formulations [24, 56, 57] and equations of the form (2.1). For the convenience of the reader, we included a derivation of Eq. (2.1) starting from the primal Lippmann–Schwinger equation in elasticity, see Appendix. A.

For a two-phase material with stiffnesses $\mathbb {C}_1$ and $\mathbb {C}_2$, distributed with volume fractions $\phi _1$ and $\phi _2 \equiv 1 - \phi _1$, respectively, the formula (2.1) simplifies as follows

2.6 $$\begin{aligned} \left[ (\mathbb {C}^{\text {eff}} - \mathbb {C}^0)^{-1} + \hat{\mathbb {\Gamma }}^0({ n})\right] ^{-1} = \phi _1 \, \left[ (\mathbb {C}_1 - \mathbb {C}^0)^{-1} + \hat{\mathbb {\Gamma }}^0({ n})\right] ^{-1} + \phi _2 \, \left[ (\mathbb {C}_2 - \mathbb {C}^0)^{-1} + \hat{\mathbb {\Gamma }}^0({ n})\right] ^{-1}. \end{aligned}$$

In case the tensor difference $\mathbb {C}_1 - \mathbb {C}_2$ is invertible, it is actually convenient to let the reference stiffness coincide with one of the phases, e.g., $\mathbb {C}^0 \equiv \mathbb {C}_2$. Then, taking the limit $\mathbb {C}^0 \rightarrow \mathbb {C}_2$ gives rise to the formula

2.7 $$\begin{aligned} \left[ (\mathbb {C}^{\text {eff}} - \mathbb {C}_2)^{-1} + \hat{\mathbb {\Gamma }}_2({ n})\right] ^{-1} = \phi _1 \, \left[ (\mathbb {C}_1 - \mathbb {C}_2)^{-1} + \hat{\mathbb {\Gamma }}_2({ n})\right] ^{-1}, \end{aligned}$$

which may be rewritten more explicitly in the form [12, §9.4]

2.8 $$\begin{aligned} \phi _1 \, (\mathbb {C}^{\text {eff}} - \mathbb {C}_2)^{-1} = (\mathbb {C}_1 - \mathbb {C}_2)^{-1} + (1-\phi _1) \, \hat{\mathbb {\Gamma }}_2({ n}). \end{aligned}$$

This expression is a convenient starting point for providing an explicit form for the effective stiffness of a coated laminate with core $\mathbb {C}_1$ and coating $\mathbb {C}_2$, i.e., by repeatedly laminating the second material into the already obtained material (see Fig. 1).

For the work at hand, we are interested in the case of *degenerate* laminates. To treat such specialities, we rewrite Eq. (2.8) by multiplying with the prefactor $\phi _1^{-1}$, inverting the resulting relation and factoring out the inverse stiffness tensor difference $(\mathbb {C}_1-\mathbb {C}_2)^{-1}$ inside the brackets. We obtain

2.9 $$\begin{aligned} \mathbb {C}^{\text {eff}} = \mathbb {C}_2 + \phi _1 \,\left[ {{\,\mathrm{\text {Id}}\,}}+ (1-\phi _1) \, (\mathbb {C}_1 - \mathbb {C}_2):\hat{\mathbb {\Gamma }}_2({ n})\right] ^{-1}:(\mathbb {C}_1 - \mathbb {C}_2). \end{aligned}$$

The formula (2.9) does not require the difference $\mathbb {C}_1 - \mathbb {C}_2$ to be regular. However, the seemingly more complicated term

2.10 $$\begin{aligned} {{\,\mathrm{\text {Id}}\,}}+ (1-\phi _1) \, (\mathbb {C}_1 - \mathbb {C}_2):\hat{\mathbb {\Gamma }}_2({ n}) \end{aligned}$$

needs to be invertible. In Sect. 3, we show that this term is indeed invertible for a positive volume fraction $\phi _1 > 0$ even in the case where the stiffness $\mathbb {C}_1$ is only positive semi-definite. To simplify matters further, we non-dimensionalize Eq. (2.9) by introducing the dimensionless quantities

2.11 $$\begin{aligned} \tilde{\mathbb {C}}^{\text {eff}} = \mathbb {C}_2^{-\frac{1}{2}} : \mathbb {C}^{\text {eff}} : \mathbb {C}_2^{-\frac{1}{2}}, \end{aligned}$$

2.12 $$\begin{aligned} \tilde{\mathbb {C}}_1 = \mathbb {C}_2^{-\frac{1}{2}} : \mathbb {C}_1 : \mathbb {C}_2^{-\frac{1}{2}}, \end{aligned}$$

2.13 $$\begin{aligned} \tilde{\mathbb {\Gamma }}_2({ n}) = \mathbb {C}_2^{\frac{1}{2}} : \hat{\mathbb {\Gamma }}_2({ n}) : \mathbb {C}_2^{\frac{1}{2}}, \end{aligned}$$

which transforms Eq. (2.9) into the expression

2.14 $$\begin{aligned} \tilde{\mathbb {C}}^{\text {eff}} = {{\,\mathrm{\text {Id}}\,}}+ \phi _1 \,\left[ {{\,\mathrm{\text {Id}}\,}}+ (1-\phi _1) \, (\tilde{\mathbb {C}}_1 - {{\,\mathrm{\text {Id}}\,}}):\tilde{\mathbb {\Gamma }}_2({ n})\right] ^{-1}:(\tilde{\mathbb {C}}_1 - {{\,\mathrm{\text {Id}}\,}}). \end{aligned}$$

Then, the transformed tensor (2.13) is an orthogonal projector on the space $\text {Sym}(d)$ of symmetric d×d tensors with respect to the Frobenius inner product. The projector (2.13) actually projects onto the vector space

2.15 $$\begin{aligned} \text {im}\left( {\tilde{\mathbb {\Gamma }}_2({ n})}\right) = \left\{ \mathbb {C}_2^{\frac{1}{2}} : \left( { u}\otimes _s { n}\right) \, \bigg | \, { u}\in \mathbb {R}^d \right\} . \end{aligned}$$

Alternatively, the operator $\hat{\mathbb {\Gamma }}_2({ n}): \mathbb {C}_2$ may be regarded as an orthogonal projector onto the subspace

2.16 $$\begin{aligned} \text {im}\left( {\hat{\mathbb {\Gamma }}_2({ n})}\right) = \left\{ { u}\otimes _s { n}\, \bigg | \, { u}\in \mathbb {R}^d \right\} \subseteq \text {Sym}(d) \end{aligned}$$

w.r.t. the $\mathbb {C}_2$-weighted Frobenius inner product

2.17 $$\begin{aligned} \text {Sym}(d) \times \text {Sym}(d) \ni ({\varepsilon }_1, {\varepsilon }_2) \mapsto {\varepsilon }_1 : \mathbb {C}_2 : {\varepsilon }_2. \end{aligned}$$

We will use either of the equivalent formulations, depending on which one is more convenient.

Equation (2.1) may also be rewritten in dual form

2.18 $$\begin{aligned} \left[ (\mathbb {D}^{\text {eff}} - \mathbb {D}^0)^{-1} + \hat{\mathbb {\Delta }}^0({ n})\right] ^{-1} = \left\langle {\left[ (\mathbb {D}- \mathbb {D}^0)^{-1} + \hat{\mathbb {\Delta }}^0({ n})\right] ^{-1}} \right\rangle , \end{aligned}$$

involving the compliance tensors

2.19 $$\begin{aligned} \mathbb {D}^{\text {eff}} = \left( \mathbb {C}^{\text {eff}} \right) ^{-1}, \quad \mathbb {D}^0 = \left( \mathbb {C}^0\right) ^{-1} \quad \text {and} \quad \mathbb {D}= \mathbb {C}^{-1} \end{aligned}$$

as well as the dual Eshelby–Green operator [56]

2.20 $$\begin{aligned} \hat{\mathbb {\Delta }}^0({ n}) = \mathbb {C}^0 - \mathbb {C}^0:\hat{\mathbb {\Gamma }}^0({ n}):\mathbb {C}^0. \end{aligned}$$

The dual formulation (2.18) naturally handles rigid phases, as the local compliance $\mathbb {D}$ may be set to zero in specific locations. As for the primal formulation (2.6), two-phase laminates lead to an expression

2.21 $$\begin{aligned} \mathbb {D}^{\text {eff}} = \mathbb {D}_2 + \phi _1 \,\left[ {{\,\mathrm{\text {Id}}\,}}+ (1-\phi _1) \, (\mathbb {D}_1 - \mathbb {D}_2):\hat{\mathbb {\Delta }}_2({ n})\right] ^{-1}:(\mathbb {D}_1 - \mathbb {D}_2). \end{aligned}$$

which may be rewritten in the abstract form (3.4). The image of the dual operator (2.20) computes as

2.22 $$\begin{aligned} \text {im}\left( {\hat{\mathbb {\Delta }}_2({ n})}\right) = \left\{ \boldsymbol{\tau }\in \text {Sym}(d) \, \bigg | \, \boldsymbol{\tau }\cdot { n}= \boldsymbol{0} \right\} . \end{aligned}$$

### Thermal conductivity

For a laminate material with local conductivity tensors $\boldsymbol{ A}$, the effective thermal conductivity tensor $\boldsymbol{ A}^{\text {eff}}$ computes via the equation [12, §9]

2.23 $$\begin{aligned} \left[ (\boldsymbol{ A}^{\text {eff}} - \boldsymbol{ A}^0)^{-1} + \hat{\boldsymbol{\Gamma }}^0({ n})\right] ^{-1} = \left\langle {\left[ (\boldsymbol{ A}- \boldsymbol{ A}^0)^{-1} + \hat{\boldsymbol{\Gamma }}^0({ n})\right] ^{-1}} \right\rangle \end{aligned}$$

for a reference conductivity $\boldsymbol{ A}^0$ and the second-order Eshelby–Green operator

2.24 $$\begin{aligned} \hat{\boldsymbol{\Gamma }}^0({ n}) = \frac{{ n}\otimes { n}}{{ n}\cdot \boldsymbol{ A}^0 { n}}. \end{aligned}$$

For a two-phase conducting laminate, the formula

2.25 $$\begin{aligned} \boldsymbol{ A}^{\text {eff}} = \boldsymbol{ A}_2 + \phi _1 \,\left[ {{\,\mathrm{\text {Id}}\,}}+ (1-\phi _1) \, (\boldsymbol{ A}_1 - \boldsymbol{ A}_2):\hat{\boldsymbol{\Gamma }}_2({ n})\right] ^{-1}:(\boldsymbol{ A}_1 - \boldsymbol{ A}_2) \end{aligned}$$

holds, similar to elasticity (2.9). In particular, a dimensionless representation of the conductivity tensors and of the Eshelby–Green operator (2.24) may be derived in the same manner as in (2.11)–(2.13).

Equivalent to Eq. (2.18) is the lamination formula

2.26 $$\begin{aligned} \left[ ({ R}^{\text {eff}} - { R}^0)^{-1} + \hat{\boldsymbol{\triangle }}^0({ n})\right] ^{-1} = \left\langle {\left[ ({ R}- { R}^0)^{-1} + \hat{\boldsymbol{\triangle }}^0({ n})\right] ^{-1}} \right\rangle , \end{aligned}$$

involving the thermal resistance tensors ${ R}$, a reference resistance ${ R}^0$ and the dual Eshelby–Green operator

2.27 $$\begin{aligned} \hat{\boldsymbol{\triangle }}^0({ n}) = \boldsymbol{ A}^0 - \boldsymbol{ A}^0 \hat{\boldsymbol{\Gamma }}^0({ n}) \boldsymbol{ A}^0. \end{aligned}$$

## Coated laminates in an abstract setting

### Simple two-phase lamination

We consider a Hilbert space *H* with fixed inner product ${\left\langle {\cdot },{\cdot }\right\rangle }$. We call a bounded linear mapping P∈L(H) an *orthogonal projector* if it is idempotent,

3.1 $$\begin{aligned} P^2 = P, \end{aligned}$$

and self-adjoint,

3.2 $$\begin{aligned} P^* = P. \end{aligned}$$

Here, $P^*$ denotes the adjoint of the projector *P* with respect to the underlying Hilbert space inner product. Equivalently, a projector *P* is orthogonal if and only if the Pythagorean identity

3.3 $$\begin{aligned} \Vert x\Vert ^2 = \Vert Px\Vert ^2 + \Vert Qx\Vert ^2, \quad Q = {{\,\mathrm{\text {Id}}\,}}-P, \end{aligned}$$

holds for all vectors x∈V, where $\Vert \cdot \Vert$ denotes the natural norm on the Hilbert space *H* and ${{\,\mathrm{\text {Id}}\,}}$ is the identity operator on *V*.

Suppose a self-adjoint and positive semi-definite operator $A_0$ is given, together with an orthogonal projector *P* and a real number $\phi \in (0,1]$. Then, we define the laminated operator $A_1$ via

3.4 $$\begin{aligned} A_1 = {{\,\mathrm{\text {Id}}\,}}+ \phi \left[ {{\,\mathrm{\text {Id}}\,}}+ (1-\phi ) (A_0 - {{\,\mathrm{\text {Id}}\,}})P \right] ^{-1} (A_0 - {{\,\mathrm{\text {Id}}\,}}). \end{aligned}$$

Equation (3.4) encompasses the effective elastic and thermal properties of coated laminates, in both the primal and the dual setting, compare Eq. (2.14), Eq. (2.21) and Eq. (2.25). The following statements hold:

1. The formula (3.4) is well defined, i.e., the bounded linear operator
  3.5 $$\begin{aligned} B = {{\,\mathrm{\text {Id}}\,}}+ (1-\phi ) (A_0 - {{\,\mathrm{\text {Id}}\,}})P \end{aligned}$$
  is invertible, see Appendix B.1.
2. The operator (3.4) is self-adjoint, i.e., the identity
  3.6 $$\begin{aligned} A_1^* = A_1 \end{aligned}$$
  holds, see Appendix B.2.
3. The operator (3.4) is positive semi-definite (or monotone), i.e., the inequality
  3.7 $$\begin{aligned} {\left\langle {x},{A_1x}\right\rangle } \ge 0 \quad \text {holds for all} \quad x \in H. \end{aligned}$$
  If the original operator $A_0$ is positive definite, i.e., there is a positive constant $\alpha _0 \in \mathbb {R}_{>0}$, s.t. the inequality
  3.8 $$\begin{aligned} {\left\langle {x},{A_0x}\right\rangle } \ge \alpha _0\,\Vert x\Vert ^2, \quad x \in H, \end{aligned}$$
  holds, the operator (3.4) is also positive definite (with a possibly different positive constant $\alpha _1$). In case the operator $A_0$ is strictly monotone, i.e., it is monotone (3.7) and satisfies the inequality
  3.9 $$\begin{aligned} {\left\langle {x},{A_0x}\right\rangle } > 0 \quad \text {for all} \quad x \in H \backslash \{0\}, \end{aligned}$$
  the operator $A_1$ is strictly monotone, as well. We refer to Appendix B.3 for the details.

A few remarks are in order.

1. In case the operator $A_0 - {{\,\mathrm{\text {Id}}\,}}$ is invertible, the formula (3.4) is equivalent to the expression
  3.10 $$\begin{aligned} \phi \left( A_1 -{{\,\mathrm{\text {Id}}\,}}\right) ^{-1} = (A_0 - {{\,\mathrm{\text {Id}}\,}})^{-1} + (1-\phi )P, \end{aligned}$$
  which is typically used in the literature, see Milton [12, §9.4] and equation (2.8) in the manuscript. With numerical stability in mind, we consider the more general case (3.4).
2. Every uniformly positive-definite operator (3.8) is also strictly monotone, while the converse does not hold in general. However, in case the Hilbert space *H* is finite-dimensional—which is true for the applications in Sect. 4—the associated quadratic form attains a positive minimum on the unit sphere, so that every strictly monotone linear operator is also strongly monotone.

### Coated laminates

We consider an iterative process of sequential (coated) lamination, i.e., for a given self-adjoint and positive semi-definite operator $A_0$, together with a sequence $(\phi _i,P_i)$ comprising at each index *i* a volume fraction $\phi _i \in (0,1]$ and an orthogonal projector $P_i$, we build the operators

3.11 $$\begin{aligned} A_{m} = {{\,\mathrm{\text {Id}}\,}}+ \phi _{m} \left[ {{\,\mathrm{\text {Id}}\,}}+ (1 - \phi _{m}) (A_{m-1} - {{\,\mathrm{\text {Id}}\,}}) P_{m}\right] ^{-1} (A_{m-1} - {{\,\mathrm{\text {Id}}\,}}), \quad m=1,2,\ldots , \end{aligned}$$

in a recursive fashion. By the results of Sect. 3.1, the construction (3.11) is well defined and gives rise to a sequence of $(A_m)$ of self-adjoint and positive semi-definite operators.

It was noticed [12, §9.4] in the non-degenerate case (3.4) that the recursive formula (3.11) may be equivalently rewritten in an explicit form. For the degenerate case which we are considering, the following *explicit* formula holds:

3.12 $$\begin{aligned} A_m = {{\,\mathrm{\text {Id}}\,}}+ \prod _{i=1}^m \phi _i \left[ {{\,\mathrm{\text {Id}}\,}}+ (A_0 - {{\,\mathrm{\text {Id}}\,}})\sum _{i=1}^m (1-\phi _i) \prod _{j=1}^{i-1} \phi _j P_i\right] ^{-1}(A_0 - {{\,\mathrm{\text {Id}}\,}}). \end{aligned}$$

The validity of this statement follows directly by mathematical induction, see Appendix B.4. A key result here is the invertibility of the operator

3.13 $$\begin{aligned} B_m = {{\,\mathrm{\text {Id}}\,}}+ (A_0 - {{\,\mathrm{\text {Id}}\,}})\sum _{i=1}^m (1-\phi _i) \prod _{j=1}^{i-1} \phi _j P_i, \end{aligned}$$

established in Appendix B.5. Actually, it is more convenient to recast the formula (3.11) in the form

3.14 $$\begin{aligned} A_m = {{\,\mathrm{\text {Id}}\,}}+ f_m \left[ {{\,\mathrm{\text {Id}}\,}}+ (1-f_m)(A_0 - {{\,\mathrm{\text {Id}}\,}})\sum _{i=1}^m c_{i,m} P_i\right] ^{-1}(A_0 - {{\,\mathrm{\text {Id}}\,}}) \end{aligned}$$

with the scalar coefficients

3.15 $$\begin{aligned} f_m = \prod _{i=1}^m \phi _i \quad \text {and} \quad c_{i,m} = \frac{(1-\phi _i) \prod _{j=1}^{i-1} \phi _j}{1-f_m}. \end{aligned}$$

Notice that the coefficients $c_{i,m}$ are non-degenerate and sum to unity. The latter may be seen directly, inspecting the telescopic sum

3.16 $$\begin{aligned}&\sum _{i=1}^m \prod _{j=1}^{i-1} (1-\phi _i) \phi _j = 1 - \phi _1 + (1-\phi _2)\phi _1 + \ldots + (1-\phi _m)\phi _1\phi _2 \cdots \phi _{m-1}\nonumber \\&\quad = 1 - \phi _1 + \phi _1 - \phi _1\phi _2 \pm \ldots + \phi _1\phi _2 \cdots \phi _{m-2} - \phi _1\phi _2 \cdots \phi _{m-1} + \phi _1\phi _2 \cdots \phi _{m-1} - \phi _1\phi _2 \cdots \phi _{m}\nonumber \\&\quad = 1 - \prod _{i=1}^m \phi _i \equiv 1 - f_m. \end{aligned}$$

As a consequence, the operator

3.17 $$\begin{aligned} C_m = \sum _{i=1}^m c_{i,m} P_i \end{aligned}$$

turns out to be a convex combination of projectors.

### Non-degeneracy of coated laminates with a degenerate core

In this section, we study the abstract coating process (3.11) under the condition that the starting operator

3.18 $$\begin{aligned} A_0 = 0 \end{aligned}$$

vanishes. For the physical examples treated in Sects. 2 and 4, this condition corresponds to the case that the material properties either vanish or are infinite (in a dual formulation). Section 3.2 establishes several properties assuming a self-adjoint and positive semi-definite operator $A_0$ only, i.e., encompassing a vanishing original operator as a special case.

The operators $(A_m)$ constructed via Eq. (3.11) turn out to be self-adjoint and positive semi-definite. Moreover, in the special case (3.18), the representation formula (3.14) becomes

3.19 $$\begin{aligned} A_m = {{\,\mathrm{\text {Id}}\,}}- f_m \left[ {{\,\mathrm{\text {Id}}\,}}- (1-f_m)\,C_m\right] ^{-1} \end{aligned}$$

with the operator $C_m$ defined in Eq. (3.17). From physical considerations, one might expect that the operator $A_m$ eventually becomes non-degenerate provided a sufficient number of lamination steps were applied.

As a key result of this article, we establish the equivalence of the following statements:

1. The operator (3.19) with $f_m \in (0,1)$ and $c_{i,m}>0$ is invertible.
2. The subspaces
  3.20 $$\begin{aligned} V_i = \left\{ x \in H\, \bigg | \, x = P_i x\right\} , \quad i=1,2,\ldots ,m, \end{aligned}$$
  which the operators $P_i$ project onto have a trivial intersection
  3.21 $$\begin{aligned} \bigcap _{i=1}^m V_i = \{ 0 \}. \end{aligned}$$

This statement follows from two observations. For a start, a vector x∈H lies in the kernel of the operator (3.19) if and only if the equation

3.22 $$\begin{aligned} C_m x = x \end{aligned}$$

holds, i.e., the vector *x* is an eigenvector of the self-adjoint operator (3.17) for the eigenvalue 1. This statement follows via simple algebraic manipulation, see Appendix B.6.

The second observation is the explicit characterization

3.23 $$\begin{aligned} C_m x = x \quad \text {if and only if} \quad x = P_i \, x \quad \text {for all} \quad i=1,2,\ldots ,m, \end{aligned}$$

of the eigenvectors (3.22). In case the projectors $P_i$ are mutually orthogonal, i.e., satisfy the property

3.24 $$\begin{aligned} P_i P_j = 0 \quad \text {for} \quad i\ne j, \end{aligned}$$

the statement (3.23) is trivial. We consider the more general case of non-orthogonal subspaces in Appendix B.7.

The two observations (3.22) and (3.23) imply the validity of characterization (3.21) of the invertibility of the operator (3.19).

## Applications

### Linear elasticity

We consider a coated laminate (3.14) with *m* laminations, each of them taken with a non-trivial volume fraction $\phi _i \in (0,1)$ (i=1,2,…,m) and with associated directions ${ n}_i$ (i=1,2,…,m) of lamination. We restrict to d=3 dimensions. Then, the following hold:

1. In case the core stiffness vanishes and the coating stiffness $\mathbb {C}_c$ is non-degenerate, the effective stiffness $\mathbb {C}^{\text {eff}}_m$ of the coated laminate, governed by the equation (3.19)
  4.1 $$\begin{aligned} \mathbb {C}^{\text {eff}}_m = \mathbb {C}_c - f_m \left[ \left( \mathbb {C}_c\right) ^{-1} - (1-f_m)\sum _{i=1}^m c_{i,m}\,\hat{\mathbb {\Gamma }}_c({ n}_i)\right] ^{-1} \end{aligned}$$
  is non-degenerate for m=3 laminations, provided the directions ${ n}_i$ (i=1,2,3) are linearly independent. Less than m=3 coated laminations are not sufficient to generate a non-degenerate stiffness.
2. For a rigid core, i.e., with vanishing compliance, and a non-degenerate coating stiffness $\mathbb {C}_c$, the effective compliance $\mathbb {D}^{\text {eff}}_m$ of the coated laminate, satisfying the relation
  4.2 $$\begin{aligned} \mathbb {D}^{\text {eff}}_m = \mathbb {D}_c - f_m \left[ \left( \mathbb {D}_c\right) ^{-1} - (1-f_m)\sum _{i=1}^m c_{i,m}\,\hat{\mathbb {\Delta }}_c({ n}_i)\right] ^{-1}, \end{aligned}$$
  is non-degenerate for m=3 laminations, provided the directions ${ n}_i$ (i=1,2,3) are linearly independent. Less than m=3 coated laminations do not produce a non-degenerate compliance tensor.

Let us discuss the first item. By the characterization (3.21), the stiffness (4.1) is non-degenerate if and only if the images of the Eshelby–Green operators $\hat{\mathbb {\Gamma }}_c({ n}_i)$ have a trivial intersection

4.3 $$\begin{aligned} \bigcap _{i=1}^m \text {im}\left( {\hat{\mathbb {\Gamma }}_c({ n}_i)}\right) = \{ \boldsymbol{0} \}. \end{aligned}$$

Due to the explicit characterization (2.16), which takes the form

4.4 $$\begin{aligned} \text {im}\left( {\hat{\mathbb {\Gamma }}_c({ n}_i)}\right) = \left\{ { u}\otimes _s { n}_i \, \bigg | \, { u}\in \mathbb {R}^d \right\} \subseteq \text {Sym}(d) \end{aligned}$$

in our case, we may study the validity of the condition (4.3) explicitly. Clearly, a single lamination m=1 is not enough. For m=2 laminations with non-collinear directions ${ n}_1$ and ${ n}_2$, we observe the characterization

4.5 $$\begin{aligned} \text {im}\left( {\hat{\mathbb {\Gamma }}_c({ n}_1)}\right) \cap \text {im}\left( {\hat{\mathbb {\Gamma }}_c({ n}_2)}\right) = \left\{ \alpha \, { n}_1 \otimes _s { n}_2 \, \bigg | \, \alpha \in \mathbb {R}\right\} \subseteq \text {Sym}(d), \end{aligned}$$

i.e., the intersection is not trivial. Thus, at least m=3 laminations are necessary to render the stiffness (4.1) non-degenerate. We briefly discuss the validity of the expression (4.5). As we assumed the directions ${ n}_1$ and ${ n}_2$ to be linearly independent, we may choose a third vector ${ n}_3$, s.t. the triplet $({ n}_1,{ n}_2,{ n}_3)$ constitutes a basis of the space $\mathbb {R}^3$. We introduce an inner product

4.6 $$\begin{aligned} \left\langle \sum _{i=1}^3 \alpha _i \, { n}_i, \sum _{j=1}^3 \beta _j \, { n}_j \right\rangle = \sum _{i=1}^3 \alpha _i \beta _i \end{aligned}$$

which turns the basis $({ n}_1,{ n}_2,{ n}_3)$ into an orthormal basis. Let us consider an element

4.7 $$\begin{aligned} {\varepsilon }\in \text {im}\left( {\hat{\mathbb {\Gamma }}_c({ n}_1)}\right) \cap \text {im}\left( {\hat{\mathbb {\Gamma }}_c({ n}_2)}\right) \end{aligned}$$

in the intersection. By definition (4.4), there are vectors ${ u},{ v}\in \mathbb {R}^3$, s.t., we may write

4.8 $$\begin{aligned} {\varepsilon }= { u}\otimes _s { n}_1 \quad \text {and} \quad {\varepsilon }= { v}\otimes _s { n}_2. \end{aligned}$$

Explicitly, we are therefore led to the equation

4.9 $$\begin{aligned} { u}\otimes { n}_1 + { n}_1 \otimes { u}= { v}\otimes { n}_2 + { n}_2 \otimes { v}. \end{aligned}$$

Taking the inner product (4.6) with the vector ${ n}_3$ from the left yields

4.10 $$\begin{aligned} \langle { u}, { n}_3 \rangle { n}_1 + \underbrace{\langle { n}_1,{ n}_3\rangle }_{=0} { u}= \langle { v},{ n}_3\rangle { n}_2 + \underbrace{\langle { n}_2, { n}_3 \rangle }_{=0} { v}. \end{aligned}$$

Taking the inner product (4.6) with the vector ${ n}_1$ yields

4.11 $$\begin{aligned} \langle { u}, { n}_3 \rangle \underbrace{\langle { n}_1,{ n}_1\rangle }_{=1} = \langle { v},{ n}_3\rangle \underbrace{\langle { n}_2,{ n}_1\rangle }_{=0} \quad \Leftrightarrow \quad \langle { u}, { n}_3 \rangle = 0. \end{aligned}$$

In particular, the vector ${ u}$ is a linear combination of the vectors ${ n}_1$ and ${ n}_2$ only.

We return to the representation (4.9) and take an inner product with the vector ${ n}_1$ from the left

4.12 $$\begin{aligned} \langle { u}, { n}_1 \rangle { n}_1 + \underbrace{\langle { n}_1,{ n}_1\rangle }_{=1} { u}= \langle { v},{ n}_1\rangle { n}_2 + \underbrace{\langle { n}_2, { n}_1 \rangle }_{=0} { v}. \end{aligned}$$

Taking another inner product with the vector ${ n}_1$ implies the identity

4.13 $$\begin{aligned} \langle { u}, { n}_1 \rangle \underbrace{\langle { n}_1,{ n}_1\rangle }_{=1} + \langle { u}, { n}_1 \rangle = \langle { v},{ n}_1\rangle \underbrace{\langle { n}_2, { n}_1 \rangle }_{=0} \quad \Leftrightarrow \quad 2 \, \langle { u}, { n}_1 \rangle = 0. \end{aligned}$$

Thus, the vector ${ u}$ needs to be collinear to the vector ${ n}_2$, which was to be shown (4.5).

The representation formula (4.5) reveals, in particular, that two laminations are not sufficient to produce a trivial intersection (4.3) which, in turn, is necessary and sufficient to generate a non-degenerate effective stiffness (4.1) of the coated laminate.

Let us discuss three laminations, m=3, with linearly independent directions $({ n}_1,{ n}_2,{ n}_3)$. We will also make use of the constructed inner product (4.6). An element

4.14 $$\begin{aligned} {\varepsilon }\in \text {im}\left( {\hat{\mathbb {\Gamma }}_c({ n}_1)}\right) \cap \text {im}\left( {\hat{\mathbb {\Gamma }}_c({ n}_2)}\right) \cap \text {im}\left( {\hat{\mathbb {\Gamma }}_c({ n}_3)}\right) \end{aligned}$$

in the intersection may be represented in the form

4.15 $$\begin{aligned} {\varepsilon }= \alpha _{ij} { n}_i \otimes _s { n}_j \quad \text {for some} \quad \alpha _{ij} \in \mathbb {R}\quad \text {and} \quad i<j. \end{aligned}$$

By similar arguments as before, taking the inner product with ${ n}_1$ and ${ n}_2$ in the representation

4.16 $$\begin{aligned} \alpha _{12} { n}_1 \otimes _s { n}_2 = \alpha _{13} { n}_1 \otimes _s { n}_3, \end{aligned}$$

i.e., the element (4.14) must necessarily vanish. In particular, the triple intersection (4.14) is trivial and the produced effective stiffness (4.1) is non-degenerate.

**Fig. 2:**
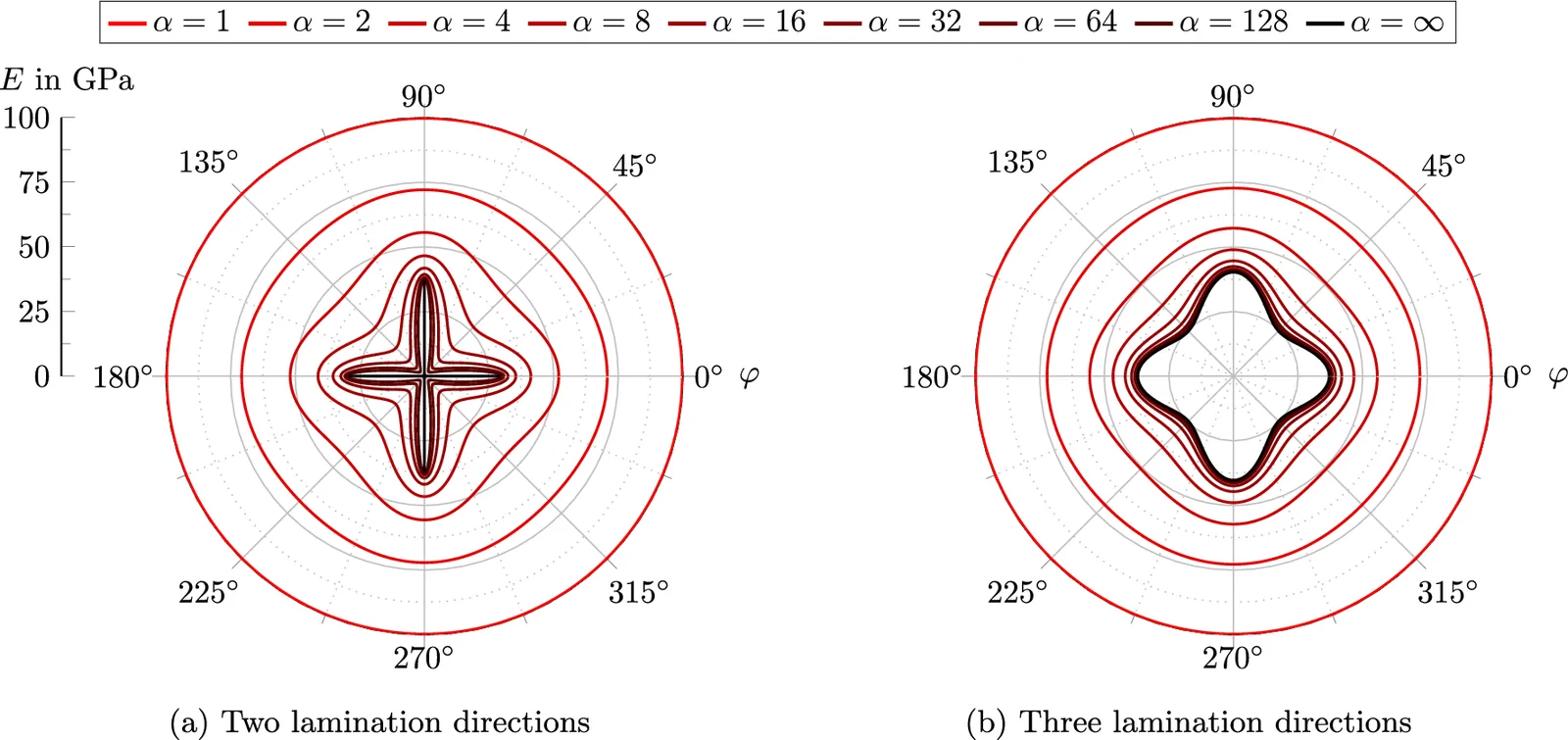
Directional Young’s modulus in the $({ e}_y,{ e}_z)$-plane of coated laminates in the soft-core limit, calculated using the coated laminate formula (3.14). The core stiffness is reduced according to $E_\textrm{core} = E_\textrm{coat}/\alpha$. **a** Two orthogonal lamination directions. **b** Three orthogonal lamination directions

The compliance case (4.2) is a bit simpler. By the explicit characterization (2.22)

4.17 $$\begin{aligned} \text {im}\left( {\hat{\mathbb {\Delta }}_c({ n}_i)}\right) = \left\{ \boldsymbol{\tau }\in \text {Sym}(d) \, \bigg | \, \boldsymbol{\tau }\cdot { n}_i = \boldsymbol{0} \right\} , \end{aligned}$$

we observe that an element $\boldsymbol{\tau }\in \text {Sym}(3)$ lies in the intersection

4.18 $$\begin{aligned} \boldsymbol{\tau }\in \bigcap _{i=1}^m \text {im}\left( {\hat{\mathbb {\Delta }}_c({ n}_i)}\right) \end{aligned}$$

if and only if the equations

4.19 $$\begin{aligned} \boldsymbol{\tau }\cdot { n}_i = \boldsymbol{0} \quad \text {hold for} \quad i=1,2,3. \end{aligned}$$

However, taking into account that the element $\boldsymbol{\tau }\in \text {Sym}(3)$ may be considered as a linear operator on $\mathbb {R}^3$, the condition (4.18) actually characterizes the zero mapping in view of the basis character of the triplet $({ n}_1,{ n}_2,{ n}_3)$. In particular, the intersection

4.20 $$\begin{aligned} \bigcap _{i=1}^3 \text {im}\left( {\hat{\mathbb {\Delta }}_c({ n}_i)}\right) = \{ \boldsymbol{0} \} \end{aligned}$$

is trivial, and the effective compliance (4.2) is non-degenerate. With a similar argument, it is also evident that two laminations are not sufficient to produce a non-degenerate compliance.

**Fig. 3:**
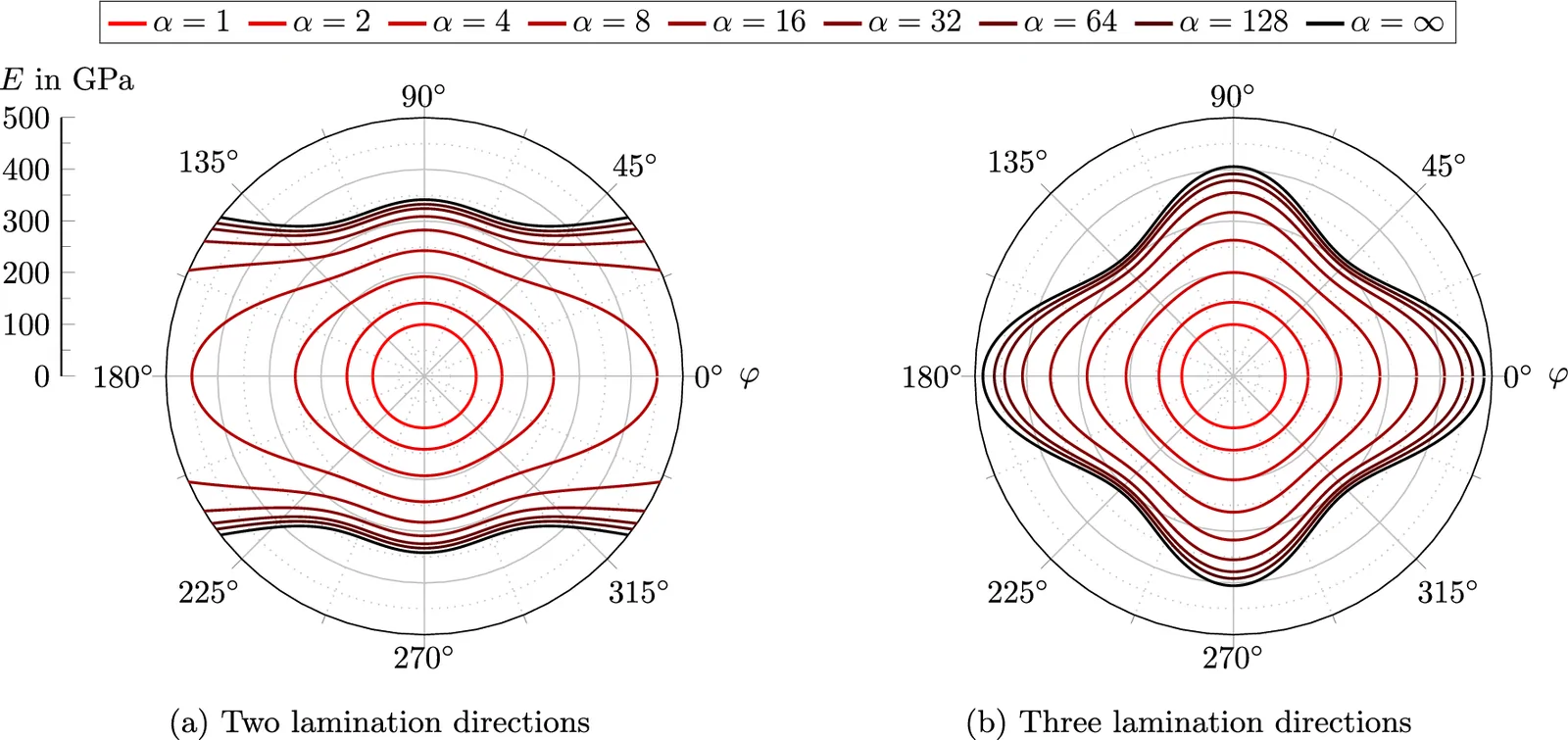
Directional Young’s modulus in the $({ e}_x,{ e}_z)$-plane of coated laminates in the rigid-core limit, calculated using the coated laminate formula (3.14). The core stiffness is increased according to $E_\textrm{core} = \alpha \,E_\textrm{coat}$. **a** Two orthogonal lamination directions. **b** Three orthogonal lamination directions

We illustrate the linear elastic cases by directional Young’s modulus plots of coated laminates in the degenerate and non-degenerate regimes, calculated using the coated laminate formula (3.14). Starting from a reference configuration where the core and coating possess identical isotropic elastic properties, we subsequently approach the respective degenerate limit via either of the rescalings $E_\textrm{core} = \alpha \,E_\textrm{coat}$ or $E_\textrm{core} = E_\textrm{coat} / \alpha$ with $\alpha \rightarrow \infty$. Throughout, the total volume fractions of core and coating are chosen to be identical. The parameters used are summarized in Table 1.

**Table 1:** Parameters for the directional Young’s modulus plots in Figs. 2 and  3

Coating Young’s modulus	$E_{\textrm{coat}}$	$100\,\textrm{GPa}$
Coating and core Poisson’s ratio	$\nu _{\textrm{coat}}$	0.30
Lamination normals (two directions)	$\{{ n}_i\}_{i=1}^2$	$\{{ e}_y,{ e}_z\}$
Lamination normals (three directions)	$\{{ n}_i\}_{i=1}^3$	$\{{ e}_y,{ e}_z,{ e}_x\}$

Figure 2 shows the soft-core case, corresponding to a vanishing core stiffness and the effective stiffness formulation (4.1). In Fig. 2a

Figure 3 illustrates the case of a rigid core, corresponding to vanishing compliance and the effective compliance formulation (4.2). In Fig. 3a,

### Thermal conductivity

We consider the same coated laminate geometry setup as in Sect. 4.1. Then, the following hold:

1. In case the core conductivity vanishes and the coating conductivity $\boldsymbol{ A}_c$ is non-degenerate, the effective conductivity $\boldsymbol{ A}^{\textrm{eff}}_m$ of the coated laminate, governed by the equation (3.19)
  4.21 $$\begin{aligned} \boldsymbol{ A}^{\textrm{eff}}_m = \boldsymbol{ A}_c - f_m \left[ \boldsymbol{ A}_c^{-1} - (1-f_m)\sum _{i=1}^m c_{i,m}\,\hat{\boldsymbol{\Gamma }}_c({ n}_i)\right] ^{-1} \end{aligned}$$
  is non-degenerate for m=2 laminations, provided the directions ${ n}_i$ (i=1,2) are not collinear. A single lamination (m=1) is not sufficient.
2. For a perfectly conducting core, i.e., with vanishing thermal resistance, and a non-degenerate coating conductivity $\boldsymbol{ A}_c$ with associated coating resistance ${ R}_c$, the effective resistance ${ R}^{\textrm{eff}}_m$ of the coated laminate, satisfying the relation
  4.22 $$\begin{aligned} { R}^{\textrm{eff}}_m = { R}_c - f_m \left[ { R}_c^{-1} - (1-f_m)\sum _{i=1}^m c_{i,m}\,\hat{\boldsymbol{\triangle }}_c({ n}_i)\right] ^{-1} \end{aligned}$$
  is non-degenerate for m=3 laminations, provided the directions ${ n}_i$ (i=1,2,3) are linearly independent. Two laminations are not sufficient to produce a non-degenerate resistance tensor.

To prove the statements above, we evaluate the characterization (3.21) for the thermal case. For the first item, the effective conductivity (4.21) is non-degenerate if and only if the images of the operators $\hat{\boldsymbol{\Gamma }}_c({ n}_i)$ have a trivial intersection, i.e.,

4.23 $$\begin{aligned} \bigcap _{i=1}^m \text {im}\left( {\hat{\boldsymbol{\Gamma }}_c({ n}_i)}\right) = \{ \boldsymbol{0} \}. \end{aligned}$$

Recalling formula (2.24) for the Eshelby–Green operator for thermal conduction,

4.24 $$\begin{aligned} \hat{\boldsymbol{\Gamma }}^0({ n}) = \frac{{ n}\otimes { n}}{{ n}\cdot \boldsymbol{ A}^0 { n}}, \end{aligned}$$

we observe that, for any vector ${ u}\in \mathbb {R}^3$,

4.25 $$\begin{aligned} \hat{\boldsymbol{\Gamma }}^0({ n})\,{ u}= \frac{{ n}({ n}\cdot { u})}{{ n}\cdot \boldsymbol{ A}^0 { n}}, \end{aligned}$$

and thus maps every vector onto a multiple of the direction ${ n}$. Conversely, the action of the operator on the direction ${ n}$ itself is nonzero, so ${ n}$ belongs to the image. Therefore, the image of the operator $\hat{\boldsymbol{\Gamma }}_c({ n}_i)$ admits the explicit characterization

4.26 $$\begin{aligned} \text {im}\left( {\hat{\boldsymbol{\Gamma }}_c({ n}_i)}\right) = \left\{ \alpha \,{ n}_i \in \mathbb {R}^3 \,\bigg |\, \alpha \in \mathbb {R}\right\} . \end{aligned}$$

Consequently, an element ${ w}\in \mathbb {R}^3$ belongs to the intersection (4.23) if and only if it is proportional to each lamination direction ${ n}_i$. Clearly, for m=1 lamination this yields the non-trivial one-dimensional space, so a single lamination is not sufficient. For m=2 laminations with non-collinear directions ${ n}_1$ and ${ n}_2$, there exists no nonzero vector ${ w}\in \mathbb {R}^3$ that is proportional to both directions; therefore,

4.27 $$\begin{aligned} \text {im}\left( {\hat{\boldsymbol{\Gamma }}_c({ n}_1)}\right) \cap \text {im}\left( {\hat{\boldsymbol{\Gamma }}_c({ n}_2)}\right) = \{ \boldsymbol{0} \}, \end{aligned}$$

holds. In particular, two non-collinear lamination directions already guarantee that the intersection in (4.23) is trivial, and adding further laminations does not alter this conclusion, proving the first item.

**Fig. 4:**
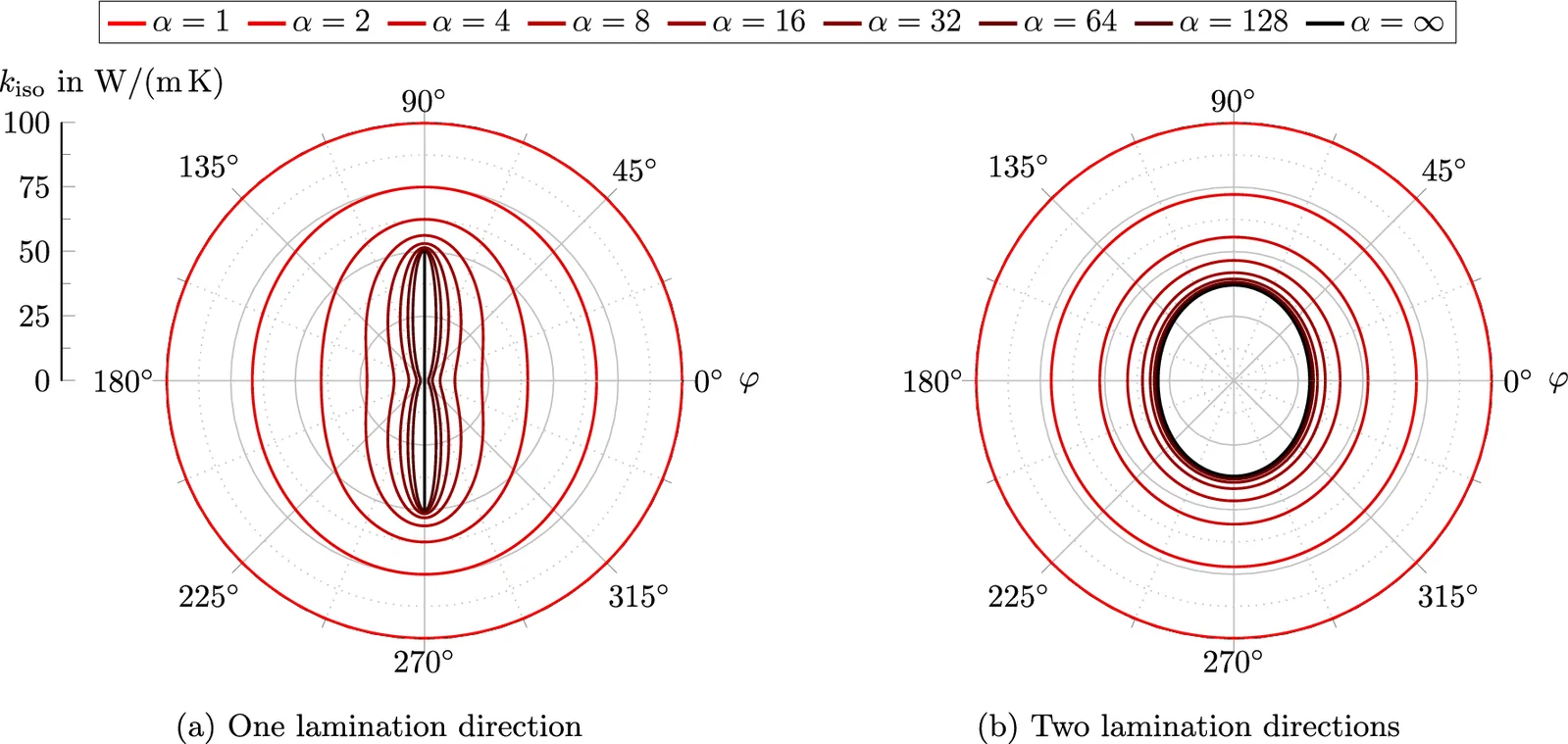
Directional thermal conductivity in the $({ e}_y,{ e}_z)$-plane of coated laminates in the soft-core limit, calculated using the coated laminate formula (3.14). The core conductivity is reduced according to $k_\textrm{core} = k_\textrm{coat}/\alpha$. **a** One lamination direction. **b** Two orthogonal lamination directions

For the second statement, the same characterization (3.21) applies. The effective resistance (4.22) is non-degenerate if and only if

4.28 $$\begin{aligned} \bigcap _{i=1}^m \text {im}\left( {\hat{\boldsymbol{\triangle }}_c({ n}_i)}\right) = \{ \boldsymbol{0} \}. \end{aligned}$$

Recalling the dual Eshelby–Green operator (2.27), we observe that its action satisfies

4.29 $$\begin{aligned} { n}\cdot \hat{\boldsymbol{\triangle }}_c({ n})\,{ u}= 0 \quad \text {for all } { u}\in \mathbb {R}^3, \end{aligned}$$

and therefore, the image admits the explicit characterization

4.30 $$\begin{aligned} \text {im}\left( {\hat{\boldsymbol{\triangle }}_c({ n}_i)}\right) = \left\{ { u}\in \mathbb {R}^3 \,\bigg |\, { u}\cdot { n}_i = 0 \right\} . \end{aligned}$$

Consequently, an element ${ u}\in \mathbb {R}^3$ belongs to the intersection (4.28) if and only if it is orthogonal to each lamination direction ${ n}_i$. For m=2 laminations with non-collinear directions ${ n}_1$ and ${ n}_2$, this condition is satisfied, for instance, by any nonzero vector parallel to the vector ${ n}_1 \times { n}_2$, so the intersection is not trivial. In contrast, for m=3 laminations with linearly independent directions, the only vector orthogonal to all three directions is the zero vector. Hence, the intersection (4.28) is trivial if and only if m≥3 holds and the lamination directions form a basis of $\mathbb {R}^3$.

**Fig. 5:**
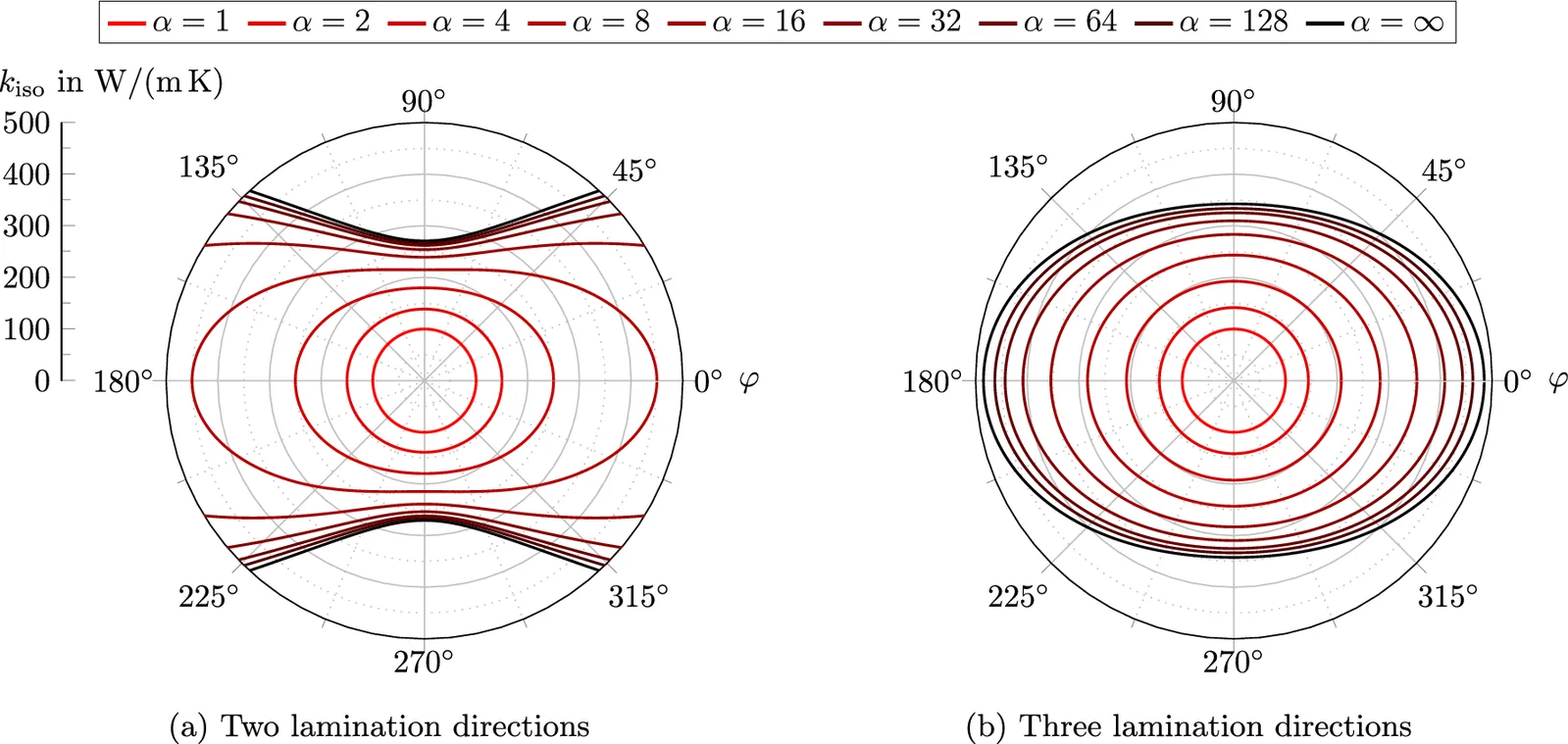
Directional thermal conductivity in the $({ e}_x,{ e}_y)$-plane of coated laminates in the rigid-core limit, calculated using the coated laminate formula (3.14). The core conductivity is increased according to $k_\textrm{core} = \alpha \,k_\textrm{coat}$. **a** Two orthogonal lamination directions. **b** Three orthogonal lamination directions

We conclude the discussion of the thermal case by illustrating the above results with directional conductivity plots of coated laminates in the degenerate and non-degenerate regimes. In all examples, the effective conductivity is evaluated using the coated laminate formula (3.14), starting from a reference configuration where core and coating possess identical isotropic conductivities and subsequently approaching the respective degenerate limits via $\alpha \rightarrow \infty$. Throughout, the total volume fractions of core and coating are chosen to be identical. The parameters used are summarized in Table 2.

**Table 2:** Parameters for the directional thermal conductivity plots in Figs. 4 and  5

Coating conductivity	$k_{\textrm{coat}}$	$100\,\mathrm {W/(m\,K)}$
Lamination normal (one direction)	$\{{ n}_i\}_{i=1}^1$	$\{{ e}_y\}$
Lamination normals (two directions)	$\{{ n}_i\}_{i=1}^2$	$\{{ e}_y,{ e}_z\}$
Lamination normals (three directions)	$\{{ n}_i\}_{i=1}^3$	$\{{ e}_y,{ e}_z,{ e}_x\}$

Figure 4 illustrates the vanishing core conductivity limit, corresponding to the effective conductivity (4.21). In Fig. 4ashows the case of two orthogonal lamination directions. Here, the effective conductivity is non-degenerate and strictly positive in all directions, yielding a polar plot without collapsed directions.

Figure 5 illustrates the limit of an infinitely conducting core, corresponding to the effective resistance formulation (4.22). In Fig. 5a, two non-collinear lamination directions are employed. Consistent with the dual characterization, the effective conductivity remains degenerate and becomes unbounded in the direction orthogonal to both lamination directions. If three mutually orthogonal lamination directions are used, as shown in Fig. 5b , the effective conductivity is non-degenerate and remains bounded in all directions.

## Conclusion

We investigated coated laminates with a degenerate core and derived precise necessary and sufficient conditions which ensure that the resulting effective operator remains non-degenerate. In contrast to classical lamination theory, which typically presupposes positive-definite material tensors and invertible tensor differences, we formulated the coating process in an abstract Hilbert space setting that naturally accommodates vanishing and infinite material coefficients. Within this framework, we established the well-posedness of the coating procedure and characterized non-degeneracy in terms of intersection properties of suitable closed linear subspaces. Rather than inferring symmetry and positivity from microstructural arguments, we derived these properties directly at the operator level. In addition, we provided a transparent derivation of the classical laminate formula from the primal Lippmann–Schwinger equation highlighting the correspondence between both formulations.

The abstract framework yields a unified treatment of thermal conductivity and linear elasticity and clarifies the mechanism by which successive laminations may restore coercivity despite degeneracy of the core, and may readily be extended to other physical fields governed by symmetric coercive operators. For an insulating core and non-degenerate coating in conductivity, two non-collinear laminations already suffice to restore strict positivity of the effective conductivity, whereas in the dual case of a perfectly conducting core, three laminations with linearly independent normals are required to obtain a non-degenerate effective resistance tensor.

The case of linear elasticity is more restrictive. For a porous core (vanishing stiffness) and non-degenerate coating stiffness, three laminations with linearly independent normals are necessary and sufficient to recover a positive-definite effective stiffness. The same requirement appears in the dual setting of a rigid core (vanishing compliance), where three independent lamination directions are needed to generate a non-degenerate effective compliance tensor.

Beyond the explicit thresholds identified for conductivity and elasticity, the analysis reveals a structural principle: The minimum number of laminations required to restore coercivity is governed by the dimension of the underlying field space. Degeneracy thus appears as a geometric property of the subspaces associated with the lamination directions, and hierarchical laminations systematically reduce the nullspaces induced by a degenerate core. These findings are directly relevant for laminate-based surrogate models such as DMNs, whose building blocks are hierarchical two-phase laminates. In particular, they provide a mathematical foundation for empirical observations in high-contrast settings and demonstrate that singular parameter regimes do not constitute a fundamental obstruction for hierarchical laminate constructions, provided a sufficient number of suitably oriented laminations is employed.

Interaction-based material networks (IMNs) were proposed as an alternative framework to DMNs that does not rely on laminate micromechanics but instead enforces the requirements of the microscopic boundary value problem at the network level [59, 60]. Systematic comparisons between DMNs and IMNs highlighted structural differences between both approaches [61]. In the literature, it was suggested that laminate-based constructions show limitations when dealing with extreme material contrast, while IMNs offer increased flexibility through a broader parameterization. In light of the present analysis, which shows that hierarchical laminations do restore coercivity even in singular limits provided a minimum number of suitably chosen layers is used, such bold statements on the limitations of classical DMNs should be reconsidered.

This perspective suggests a constructive extension of laminate-based networks: Instead of employing simple two-phase laminates as elementary building blocks, one may incorporate coated laminates with a minimum number of layers sufficient to guarantee non-degeneracy by design. Such an approach would enlarge the admissible parameter range of DMN-type architectures while retaining their micromechanical interpretability. From a broader modeling viewpoint, hierarchical coated laminates further provide a natural candidate for constructing non-degenerate composite voxels in FFT-based homogenization when standard laminate representations fail to maintain coercivity, for instance in porous or rigid media.

## Data Availability

No datasets were generated or analyzed during the current study.

## Obtaining the coated laminate formula directly from the Lippmann–Schwinger equation

We suppose that the Lippmann–Schwinger equation [24, 56, 57]

A.1 $$\begin{aligned} {\varepsilon }+ \mathbb {\Gamma }^0:(\mathbb {C}- \mathbb {C}^0):{\varepsilon }= \bar{{\varepsilon }} \end{aligned}$$

is known. In case the microstructure is a laminate with lamination direction ${ n}$, we wish to derive the following formula [12, §9.3]:

A.2 $$\begin{aligned} \left[ \left( \mathbb {C}^{\text {eff}} - \mathbb {C}^0\right) ^{-1} + \hat{\mathbb {\Gamma }}^0({ n})\right] ^{-1} = \left\langle {\left[ \left( \mathbb {C}- \mathbb {C}^0\right) ^{-1} + \hat{\mathbb {\Gamma }}^0({ n})\right] ^{-1}} \right\rangle , \end{aligned}$$

which constitutes an implicit expression for the effective stiffness $\mathbb {C}^{\text {eff}}$ of the laminate in terms of suitable volume averages. To deduce this formula, we recall the action of the Eshelby–Green operator on an arbitrary field $\boldsymbol{\tau }\in L^2(Y;\text {Sym}(d))$

A.3 $$\begin{aligned} \mathbb {\Gamma }^0:\boldsymbol{\tau }({ x}) = \sum _{\boldsymbol{\xi }\in \mathbb {Z}^d} \hat{\mathbb {\Gamma }}^0(\boldsymbol{\xi }):\hat{\boldsymbol{\tau }}(\boldsymbol{\xi }) \exp \left( \frac{2\pi i}{L} { x}\cdot \boldsymbol{\xi }\right) \end{aligned}$$

on the cube $Y=[0,L]^d$. We need two properties of the Eshelby–Green operator:

1. It vanishes on the zero frequency:
  A.4 $$\begin{aligned} \hat{\mathbb {\Gamma }}^0(\boldsymbol{0}) = \boldsymbol{0}. \end{aligned}$$
2. It is zero-homogeneous
  A.5 $$\begin{aligned} \hat{\mathbb {\Gamma }}^0(\lambda \, \boldsymbol{\xi }) = \mathbb {\Gamma }^0(\boldsymbol{\xi }), \quad \lambda \ne 0. \end{aligned}$$

Suppose that the stiffness varies only in direction ${\eta }\in \mathbb {Z}^d \backslash \{0\}$ and ${\eta }$ is “minimal” in the sense that there is no smaller multiple of ${\eta }\in \mathbb {Z}^d \backslash \{0\}$. Put differently, we suppose that the local stiffness has the form

A.6 $$\begin{aligned} \mathbb {C}({ x}) = \check{\mathbb {C}} ({\eta }\cdot { x}) \end{aligned}$$

for a one-dimensional function $\check{\mathbb {C}}$. Then, the local strain and stress fields will also have this form. Actually, we will be more interested in the stress polarization

A.7 $$\begin{aligned} \boldsymbol{\tau }= (\mathbb {C}- \mathbb {C}^0):{\varepsilon }. \end{aligned}$$

We note two things:

1. For a suitable choice of the reference material $\mathbb {C}^0$, the Lippmann–Schwinger equation may be rewritten in terms of the polarization $\boldsymbol{\tau }$
  A.8 $$\begin{aligned} \left[ \left( \mathbb {C}- \mathbb {C}^0\right) ^{-1} + \mathbb {\Gamma }^0\right] \boldsymbol{\tau }= \bar{{\varepsilon }}. \end{aligned}$$
2. As the polarization also has the form (A.6), its Fourier series may be expressed as follows
  A.9 $$\begin{aligned} \boldsymbol{\tau }({ x}) = \sum _{k \in \mathbb {Z}} \hat{\boldsymbol{\tau }}(k{\eta }) \exp \left( \frac{2k\pi i}{L} { x}\cdot {\eta }\right) , \quad { x}\in Y. \end{aligned}$$

We evaluate the action of the Eshelby–Green operator on the polarization field $\boldsymbol{\tau }$

A.10 $$\begin{aligned} \mathbb {\Gamma }^0:\boldsymbol{\tau }({ x})= &   \sum _{k \in \mathbb {Z}} \hat{\mathbb {\Gamma }}^0(k{\eta }):\hat{\boldsymbol{\tau }}(k{\eta }) \exp \left( \frac{2k\pi i}{L} { x}\cdot {\eta }\right) \nonumber \\= &   \sum _{k \in \mathbb {Z}\backslash \{0\}} \hat{\mathbb {\Gamma }}^0(k{\eta }):\hat{\boldsymbol{\tau }}(k{\eta }) \exp \left( \frac{2k\pi i}{L} { x}\cdot {\eta }\right) \nonumber \\= &   \sum _{k \in \mathbb {Z}\backslash \{0\}} \hat{\mathbb {\Gamma }}^0({\eta }):\hat{\boldsymbol{\tau }}(k{\eta }) \exp \left( \frac{2k\pi i}{L} { x}\cdot {\eta }\right) \nonumber \\= &   \hat{\mathbb {\Gamma }}^0({\eta }): \sum _{k \in \mathbb {Z}\backslash \{0\}} \hat{\boldsymbol{\tau }}(k{\eta }) \exp \left( \frac{2k\pi i}{L} { x}\cdot {\eta }\right) \nonumber \\= &   \hat{\mathbb {\Gamma }}^0({\eta }): \left[ \sum _{k \in \mathbb {Z}} \hat{\boldsymbol{\tau }}(k{\eta }) \exp \left( \frac{2k\pi i}{L} { x}\cdot {\eta }\right) - \hat{\boldsymbol{\tau }}(\boldsymbol{0})\right] \nonumber \\= &   \hat{\mathbb {\Gamma }}^0({\eta }): \left[ \boldsymbol{\tau }({ x}) - \left\langle {\boldsymbol{\tau }} \right\rangle \right] , \end{aligned}$$

where we used the properties (A.4) and (A.5), a representation argument that was also employed in related Fourier–Radon formulations [62]. With this observation at hand, we may rewrite the Lippmann–Schwinger equation (A.8)

A.11 $$\begin{aligned} \bar{{\varepsilon }} = \left( \mathbb {C}- \mathbb {C}^0\right) ^{-1}:\boldsymbol{\tau }+ \mathbb {\Gamma }^0:\boldsymbol{\tau }= \left( \mathbb {C}- \mathbb {C}^0\right) ^{-1}:\boldsymbol{\tau }+ \hat{\mathbb {\Gamma }}^0({\eta }): \left[ \boldsymbol{\tau }- \left\langle {\boldsymbol{\tau }} \right\rangle \right] , \end{aligned}$$

i.e.,

A.12 $$\begin{aligned} \left( \mathbb {C}- \mathbb {C}^0\right) ^{-1}:\boldsymbol{\tau }+ \hat{\mathbb {\Gamma }}^0({\eta }): \boldsymbol{\tau }= \bar{{\varepsilon }} + \hat{\mathbb {\Gamma }}^0({\eta }): \left\langle {\boldsymbol{\tau }} \right\rangle . \end{aligned}$$

Inverting the operator on the left-hand side, we find the expression

A.13 $$\begin{aligned} \boldsymbol{\tau }= \left[ \left( \mathbb {C}- \mathbb {C}^0\right) ^{-1} + \hat{\mathbb {\Gamma }}^0({\eta }) \right] ^{-1}: \left( \bar{{\varepsilon }} + \hat{\mathbb {\Gamma }}^0({\eta }): \left\langle {\boldsymbol{\tau }} \right\rangle \right) . \end{aligned}$$

Taking spatial averages yields

A.14 $$\begin{aligned} \left\langle {\boldsymbol{\tau }} \right\rangle = \left\langle {\left[ \left( \mathbb {C}- \mathbb {C}^0\right) ^{-1} + \hat{\mathbb {\Gamma }}^0({\eta }) \right] ^{-1}} \right\rangle : \left( \bar{{\varepsilon }} + \hat{\mathbb {\Gamma }}^0({\eta }): \left\langle {\boldsymbol{\tau }} \right\rangle \right) , \end{aligned}$$

where we used that the field on the right-hand side is homogeneous. On the other hand, by the definition of the polarization (A.7), its average may be expressed in the form

A.15 $$\begin{aligned} \left\langle {\boldsymbol{\tau }} \right\rangle = (\mathbb {C}^{\text {eff}} - \mathbb {C}^0):\bar{{\varepsilon }}, \quad \text {i.e.,} \quad \bar{{\varepsilon }} = (\mathbb {C}^{\text {eff}} - \mathbb {C}^0)^{-1}:\left\langle {\boldsymbol{\tau }} \right\rangle . \end{aligned}$$

Adding a zero yields (to mimic the right-hand side of Eq. (A.14))

A.16 $$\begin{aligned} \left[ (\mathbb {C}^{\text {eff}} - \mathbb {C}^0)^{-1} + \hat{\mathbb {\Gamma }}^0({\eta }) \right] :\left\langle {\boldsymbol{\tau }} \right\rangle = \bar{{\varepsilon }} + \hat{\mathbb {\Gamma }}^0({\eta }): \left\langle {\boldsymbol{\tau }} \right\rangle . \end{aligned}$$

Inverting the operator on the left-hand side leads to the equation

A.17 $$\begin{aligned} \left\langle {\boldsymbol{\tau }} \right\rangle = \left[ (\mathbb {C}^{\text {eff}} - \mathbb {C}^0)^{-1} + \hat{\mathbb {\Gamma }}^0({\eta }) \right] ^{-1}:\left( \bar{{\varepsilon }} + \hat{\mathbb {\Gamma }}^0({\eta }): \left\langle {\boldsymbol{\tau }} \right\rangle \right) . \end{aligned}$$

In view of Eq. (A.14), we have thus established the formula

A.18 $$\begin{aligned} \left[ (\mathbb {C}^{\text {eff}} - \mathbb {C}^0)^{-1} + \hat{\mathbb {\Gamma }}^0({\eta }) \right] ^{-1}:\left( \bar{{\varepsilon }} + \hat{\mathbb {\Gamma }}^0({\eta }): \left\langle {\boldsymbol{\tau }} \right\rangle \right) = \left\langle {\left[ \left( \mathbb {C}- \mathbb {C}^0\right) ^{-1} + \hat{\mathbb {\Gamma }}^0({\eta }) \right] ^{-1}} \right\rangle : \left( \bar{{\varepsilon }} + \hat{\mathbb {\Gamma }}^0({\eta }): \left\langle {\boldsymbol{\tau }} \right\rangle \right) . \end{aligned}$$

Using Eq. (A.15) we rewrite the term as follows

A.19 $$\begin{aligned} \begin{aligned} \bar{{\varepsilon }} + \hat{\mathbb {\Gamma }}^0({\eta }): \left\langle {\boldsymbol{\tau }} \right\rangle&= \bar{{\varepsilon }} + \hat{\mathbb {\Gamma }}^0({\eta }): (\mathbb {C}^{\text {eff}} - \mathbb {C}^0) : \bar{{\varepsilon }}\\&= ({{\,\mathrm{\text {Id}}\,}}- \hat{\mathbb {\Gamma }}({\eta })):\bar{{\varepsilon }} + \hat{\mathbb {\Gamma }}^0({\eta }): \mathbb {C}^{\text {eff}}:\bar{{\varepsilon }} \\&= \left[ ({{\,\mathrm{\text {Id}}\,}}- \hat{\mathbb {\Gamma }}({\eta })) + \hat{\mathbb {\Gamma }}^0({\eta }): \mathbb {C}^{\text {eff}}\right] :\bar{{\varepsilon }},\\ \end{aligned} \end{aligned}$$

where we use the abbreviation $\hat{\mathbb {\Gamma }}({\eta }):= \hat{\mathbb {\Gamma }}^0({\eta }):\mathbb {C}^0$. As the applied strain $\bar{{\varepsilon }}$ was arbitrary, we conclude the identity

A.20 $$\begin{aligned} \begin{aligned}&\phantom {=}\,\left[ (\mathbb {C}^{\text {eff}} - \mathbb {C}^0)^{-1} + \hat{\mathbb {\Gamma }}^0({\eta }) \right] ^{-1}:\left[ ({{\,\mathrm{\text {Id}}\,}}- \hat{\mathbb {\Gamma }}({\eta })) + \hat{\mathbb {\Gamma }}^0({\eta }): \mathbb {C}^{\text {eff}}\right] \\&\qquad {=}\, \left\langle {\left[ \left( \mathbb {C}- \mathbb {C}^0\right) ^{-1} + \hat{\mathbb {\Gamma }}^0({\eta }) \right] ^{-1}} \right\rangle :\left[ ({{\,\mathrm{\text {Id}}\,}}- \hat{\mathbb {\Gamma }}({\eta })) + \hat{\mathbb {\Gamma }}^0({\eta }): \mathbb {C}^{\text {eff}}\right] . \end{aligned} \end{aligned}$$

Thus, provided the operator

A.21 $$\begin{aligned} \mathbb {K} = \left[ ({{\,\mathrm{\text {Id}}\,}}- \hat{\mathbb {\Gamma }}({\eta })) + \hat{\mathbb {\Gamma }}^0({\eta }): \mathbb {C}^{\text {eff}}\right] \end{aligned}$$

is invertible, we thus established the identity

A.22 $$\begin{aligned} \left[ (\mathbb {C}^{\text {eff}} - \mathbb {C}^0)^{-1} + \hat{\mathbb {\Gamma }}^0({\eta }) \right] ^{-1} = \left\langle {\left[ \left( \mathbb {C}- \mathbb {C}^0\right) ^{-1} + \hat{\mathbb {\Gamma }}^0({\eta }) \right] ^{-1}} \right\rangle , \end{aligned}$$

which is precisely the sought formula (A.2) for ${ n}= {\eta }/ \Vert {\eta }\Vert$. Thus, it remains to show that the operator (A.21) is invertible. As we are in finite dimensions, the rank–nullity theorem implies that it is sufficient to show that the kernel of the operator $\mathbb {K}$ is trivial. Let us thus examine an element $\bar{{\varepsilon }} \in \text {Sym}(d)$ in the kernel of the operator $\mathbb {K}$

A.23 $$\begin{aligned} \boldsymbol{0} = \mathbb {K}:\bar{{\varepsilon }} \equiv ({{\,\mathrm{\text {Id}}\,}}- \hat{\mathbb {\Gamma }}({\eta })):\bar{{\varepsilon }} + \hat{\mathbb {\Gamma }}^0({\eta }): \mathbb {C}^{\text {eff}}:\bar{{\varepsilon }}. \end{aligned}$$

Since the reference stiffness is of the form $\mathbb {C}^0=\lambda \,{{\,\mathrm{\text {Id}}\,}}$ with λ>0, we may simplify to

A.24 $$\begin{aligned} \boldsymbol{0} = ({{\,\mathrm{\text {Id}}\,}}- \hat{\mathbb {\Gamma }}({\eta })):\bar{{\varepsilon }} + \lambda ^{-1}\hat{\mathbb {\Gamma }}({\eta }): \mathbb {C}^{\text {eff}}:\bar{{\varepsilon }}. \end{aligned}$$

As the operator $\hat{\mathbb {\Gamma }}({\eta })$ is an *orthogonal* projector, both terms

A.25 $$\begin{aligned} \boldsymbol{0}&= ({{\,\mathrm{\text {Id}}\,}}- \hat{\mathbb {\Gamma }}({\eta })):\bar{{\varepsilon }},\end{aligned}$$

A.26 $$\begin{aligned} \boldsymbol{0}&= \hat{\mathbb {\Gamma }}({\eta }): \mathbb {C}^{\text {eff}}:\bar{{\varepsilon }}, \end{aligned}$$

belong to orthogonal subspaces and hence must vanish individually. Inserting the first equation into the second yields

A.27 $$\begin{aligned} \boldsymbol{0} = \hat{\mathbb {\Gamma }}({\eta }): \mathbb {C}^{\text {eff}}:\bar{{\varepsilon }} = \hat{\mathbb {\Gamma }}({\eta }): \mathbb {C}^{\text {eff}}:\hat{\mathbb {\Gamma }}({\eta }):\bar{{\varepsilon }}, \end{aligned}$$

i.e., the quadratic form

A.28 $$\begin{aligned} 0 = \bar{{\varepsilon }}:\hat{\mathbb {\Gamma }}({\eta }): \mathbb {C}^{\text {eff}}:\hat{\mathbb {\Gamma }}({\eta }):\bar{{\varepsilon }}. \end{aligned}$$

As the effective stiffness $\mathbb {C}^{\text {eff}}$ is non-degenerate (which follows if all $\mathbb {C}$ were non-degenerate in the first place), we conclude the identity

A.29 $$\begin{aligned} \boldsymbol{0} = \hat{\mathbb {\Gamma }}({\eta }):\bar{{\varepsilon }} = \bar{{\varepsilon }}, \end{aligned}$$

where we used equation (A.25) to conclude that the kernel of the operator $\mathbb {K}$ is trivial. Thus, the operator (A.21) is invertible, and the identity (A.22) holds.

## Proofs for the abstract setting

### Invertibility of the operator in Eq. (3.5)

The purpose of this section is to show that the formula (3.4) makes sense, i.e., the bounded linear operator (3.5)

B.1 $$\begin{aligned} B = {{\,\mathrm{\text {Id}}\,}}+ (1-\phi ) (A_0 - {{\,\mathrm{\text {Id}}\,}})P \end{aligned}$$

admits a bounded inverse. Actually, this statement is a special case of the more general result established in Appendix B.5. Still, as the arguments are more elementary, we report them here.

We show the injectivity and the surjectivity of the operator (B.1) separately. For the injectivity, we study an element x∈H in the kernel of *B*, i.e., which satisfies

B.2 $$\begin{aligned} 0 = Bx \equiv x + (1 - \phi ) A_0 P x - (1-\phi ) P x. \end{aligned}$$

Taking the inner product with the vector *Px* yields

B.3 $$\begin{aligned} 0 = {\left\langle {Px},{x}\right\rangle } + (1-\phi ) {\left\langle {Px},{A_0 Px}\right\rangle } - (1-\phi ) \Vert Px\Vert ^2. \end{aligned}$$

As the operator *P* is self-adjoint (3.2), the identity

B.4 $$\begin{aligned} {\left\langle {Px},{x}\right\rangle } = \Vert Px\Vert ^2 \end{aligned}$$

holds. Therefore, we may re-arrange the terms as follows:

B.5 $$\begin{aligned} {\left\langle {Px},{x}\right\rangle } - (1-\phi ) \Vert Px\Vert ^2 = \phi \Vert Px\Vert ^2. \end{aligned}$$

Consequently, we obtain the formula

B.6 $$\begin{aligned} 0 = (1-\phi ) {\left\langle {Px},{A_0 Px}\right\rangle } + \phi \Vert Px\Vert ^2. \end{aligned}$$

As the operator $A_0$ is positive semi-definite, and the volume fraction ϕ is positive, the term $\Vert Px\Vert ^2$ must be zero. Put differently, *Px* must be the zero vector. In particular, Eq. (B.2) reveals that *x* needs to vanish. Therefore, the kernel of the operator *B* is trivial.

To study surjectivity, we take a look at the kernel of the adjoint operator

B.7 $$\begin{aligned} B^* = {{\,\mathrm{\text {Id}}\,}}+ (1-\phi ) P(A_0 - {{\,\mathrm{\text {Id}}\,}}), \end{aligned}$$

where we used that the operator *P* is orthogonal and $A_0$ is self-adjoint. An element *x* of the kernel of this operator is characterized by the equation

B.8 $$\begin{aligned} 0 = B^*x = x + (1-\phi ) P (A_0 - {{\,\mathrm{\text {Id}}\,}})x. \end{aligned}$$

Applying the complementary projector $Q = {{\,\mathrm{\text {Id}}\,}}- P$ to this equation yields

B.9 $$\begin{aligned} 0 = Qx + (1-\phi ) \underbrace{QP}_{=0} (A_0 - {{\,\mathrm{\text {Id}}\,}})x = Qx. \end{aligned}$$

Therefore, the representation

B.10 $$\begin{aligned} x = Px + Qx = Px \end{aligned}$$

is valid. Hence, we might rewrite Eq. (B.8)

B.11 $$\begin{aligned} 0 = x + (1-\phi ) P A_0 x - (1-\phi )Px = x + (1-\phi ) P A_0 P x - (1-\phi )x = (1-\phi ) P A_0 P x + \phi \, x \end{aligned}$$

An inner product with the vector *x* leads to the equation

B.12 $$\begin{aligned} 0 = (1-\phi ) {\left\langle {Px},{ A_0 P x}\right\rangle } + \phi \, \Vert x\Vert ^2. \end{aligned}$$

As the operator $A_0$ is positive semi-definite, we must have $\Vert x\Vert ^2 = 0$. Therefore, the vector *x* must vanish, and the kernel of the adjoint operator (B.7) is trivial. As a consequence, the operator *B* is surjective, concluding the derivation.

### Symmetry of the laminated operator

We would like to show that the operator (3.4)

B.13 $$\begin{aligned} A_1 = {{\,\mathrm{\text {Id}}\,}}+ \phi \left[ {{\,\mathrm{\text {Id}}\,}}+ (1-\phi ) (A_0 - {{\,\mathrm{\text {Id}}\,}})P \right] ^{-1} (A_0 - {{\,\mathrm{\text {Id}}\,}}) \end{aligned}$$

is self-adjoint. The adjoint operator

B.14 $$\begin{aligned} A_1^* = {{\,\mathrm{\text {Id}}\,}}+ \phi (A_0 - {{\,\mathrm{\text {Id}}\,}}) \left[ {{\,\mathrm{\text {Id}}\,}}+ (1-\phi ) P(A_0 - {{\,\mathrm{\text {Id}}\,}}) \right] ^{-1} \end{aligned}$$

acts on an arbitrary vector x∈H via

B.15 $$\begin{aligned} A_1^*x = x + \phi (A_0 - {{\,\mathrm{\text {Id}}\,}}) \left[ {{\,\mathrm{\text {Id}}\,}}+ (1-\phi ) P(A_0 - {{\,\mathrm{\text {Id}}\,}}) \right] ^{-1}x, \end{aligned}$$

whereas the action of the operator (B.13) reads

B.16 $$\begin{aligned} A_1x = x + \phi \left[ {{\,\mathrm{\text {Id}}\,}}+ (1-\phi ) (A_0 - {{\,\mathrm{\text {Id}}\,}})P \right] ^{-1} (A_0 - {{\,\mathrm{\text {Id}}\,}})x. \end{aligned}$$

Thus, it suffices to show that the two vectors

B.17 $$\begin{aligned} y = (A_0 - {{\,\mathrm{\text {Id}}\,}}) \left[ {{\,\mathrm{\text {Id}}\,}}+ (1-\phi ) P(A_0 - {{\,\mathrm{\text {Id}}\,}}) \right] ^{-1}x \quad \text {and} \quad z = \left[ {{\,\mathrm{\text {Id}}\,}}+ (1-\phi ) (A_0 - {{\,\mathrm{\text {Id}}\,}})P \right] ^{-1} (A_0 - {{\,\mathrm{\text {Id}}\,}})x \end{aligned}$$

coincide. We observe

B.18 $$\begin{aligned} \begin{aligned} \left[ {{\,\mathrm{\text {Id}}\,}}+ (1-\phi ) (A_0 - {{\,\mathrm{\text {Id}}\,}})P \right] y&= \left[ {{\,\mathrm{\text {Id}}\,}}+ (1-\phi ) (A_0 - {{\,\mathrm{\text {Id}}\,}})P \right] (A_0 - {{\,\mathrm{\text {Id}}\,}}) \left[ {{\,\mathrm{\text {Id}}\,}}+ (1-\phi ) P(A_0 - {{\,\mathrm{\text {Id}}\,}}) \right] ^{-1}x\\&= \left[ A_0 - {{\,\mathrm{\text {Id}}\,}}+ (1-\phi ) (A_0 - {{\,\mathrm{\text {Id}}\,}})P(A_0 - {{\,\mathrm{\text {Id}}\,}}) \right] \left[ {{\,\mathrm{\text {Id}}\,}}+ (1-\phi ) P(A_0 - {{\,\mathrm{\text {Id}}\,}}) \right] ^{-1}x\\&= (A_0 - {{\,\mathrm{\text {Id}}\,}}) \left[ {{\,\mathrm{\text {Id}}\,}}+ P(A_0 - {{\,\mathrm{\text {Id}}\,}}) \right] \left[ {{\,\mathrm{\text {Id}}\,}}+ (1-\phi ) P(A_0 - {{\,\mathrm{\text {Id}}\,}}) \right] ^{-1}x\\&= (A_0 - {{\,\mathrm{\text {Id}}\,}})x, \end{aligned} \end{aligned}$$

i.e., we conclude

B.19 $$\begin{aligned} y = \left[ {{\,\mathrm{\text {Id}}\,}}+ (1-\phi ) (A_0 - {{\,\mathrm{\text {Id}}\,}})P \right] ^{-1}(A_0 - {{\,\mathrm{\text {Id}}\,}})x \equiv z, \end{aligned}$$

which was to be shown.

### Positivity of the laminated operator

We would like to show that the self-adjoint operator (3.4)

B.20 $$\begin{aligned} A_1 = {{\,\mathrm{\text {Id}}\,}}+ \phi \left[ {{\,\mathrm{\text {Id}}\,}}+ (1-\phi ) (A_0 - {{\,\mathrm{\text {Id}}\,}})P \right] ^{-1} (A_0 - {{\,\mathrm{\text {Id}}\,}}) \end{aligned}$$

is positive semi-definite, in general, and positive definite/strictly monotone if the original operator $A_0$ is positive definite/strictly monotone and ϕ is positive. The key equation is the following representation formula

B.21 $$\begin{aligned} {\left\langle {x},{A_1 x}\right\rangle } = (1-\phi ) \Vert y + P(A_0 - {{\,\mathrm{\text {Id}}\,}}) y\Vert ^2 + \phi \, {\left\langle {y},{A_0 y}\right\rangle }\quad \text {for any} \quad x \in H \quad \text {with} \quad y = (B^*)^{-1}x. \end{aligned}$$

Here, the operator

B.22 $$\begin{aligned} B^* = {{\,\mathrm{\text {Id}}\,}}+ (1-\phi ) P(A_0 - {{\,\mathrm{\text {Id}}\,}}) \end{aligned}$$

is the adjoint of the operator *B* defined in Eq. (3.5). The invertibility of the operator *B* (and thus of $B^*$) is shown in Appendix B.1.

Assuming the validity of the equation (B.21), it is immediate to see that the operator $A_1$ is positive semi-definite. Indeed, all the terms appearing on the right-hand side of Eq. (B.21) are nonnegative, as we assumed the operator $A_0$ to be positive semi-definite. In case $A_0$ is strictly monotone (3.9), under the condition

B.23 $$\begin{aligned} {\left\langle {x},{A_1 x}\right\rangle } = 0, \end{aligned}$$

the equation

B.24 $$\begin{aligned} {\left\langle {y},{A_0 y}\right\rangle } = 0 \end{aligned}$$

follows provided the volume fraction ϕ is positive. By the strict monotoncitiy of the operator $A_0$, the vector *y* must vanish. By definition (B.21), $x = B^* y$, the vector *x* must vanish as well. Therefore, the operator $A_1$ is strictly monotone.

Suppose the operator $A_0$ is positive definite, i.e., there is a positive constant $\alpha _0$, s.t. the inequality

B.25 $$\begin{aligned} {\left\langle {x},{A_1 x}\right\rangle } \ge \alpha _0 \, \Vert x\Vert ^2, \quad x \in H, \end{aligned}$$

holds. By the representation formula (B.21), we observe for x∈H and $y = (B^*)^{-1}x$

B.26 $$\begin{aligned} {\left\langle {x},{A_1 x}\right\rangle } = (1-\phi ) \Vert y + P(A_0 - {{\,\mathrm{\text {Id}}\,}}) y\Vert ^2 + \phi \, {\left\langle {y},{A_0 y}\right\rangle } \ge {\left\langle {y},{A_0 y}\right\rangle } \ge \alpha _0 \Vert y\Vert ^2. \end{aligned}$$

By definition of the operator norm, the identity $x = B^* y$ implies the estimate

B.27 $$\begin{aligned} \Vert x\Vert \le \Vert B^*\Vert \, \Vert y\Vert , \end{aligned}$$

i.e., we are led to the desired estimate (3.8)

B.28 $$\begin{aligned} {\left\langle {x},{A_1 x}\right\rangle } \ge \alpha _0 \Vert y\Vert ^2 \ge \frac{\alpha _0}{\Vert B^*\Vert ^2} \, \Vert x\Vert ^2 \end{aligned}$$

for the operator $A_1$ with the constant $\alpha _1 = \alpha _0 / \Vert B^*\Vert ^2$.

It remains to show the key representation formula (B.21). Therefore, we express the operator $A_1$ in the form

B.29 $$\begin{aligned} \begin{aligned} A_1&= {{\,\mathrm{\text {Id}}\,}}+ \phi \left[ {{\,\mathrm{\text {Id}}\,}}+ (1-\phi ) (A_0 - {{\,\mathrm{\text {Id}}\,}})P \right] ^{-1} (A_0 - {{\,\mathrm{\text {Id}}\,}})\\&= {{\,\mathrm{\text {Id}}\,}}+ \phi \, B^{-1}(A_0 - {{\,\mathrm{\text {Id}}\,}})\\  &= B^{-1}[B + \phi (A_0 - {{\,\mathrm{\text {Id}}\,}})]. \end{aligned} \end{aligned}$$

Then, we observe

B.30 $$\begin{aligned} \begin{aligned} {\left\langle {x},{A_1x}\right\rangle }&= {\left\langle {x},{B^{-1}[B + \phi (A_0 - {{\,\mathrm{\text {Id}}\,}})]x}\right\rangle }\\&= {\left\langle {\left( B^{-1}\right) ^* x},{[B + \phi (A_0 - {{\,\mathrm{\text {Id}}\,}})]x}\right\rangle }\\&= {\left\langle {y},{[B + \phi (A_0 - {{\,\mathrm{\text {Id}}\,}})]B^*y}\right\rangle }\\&= \Vert B^*y\Vert ^2 + \phi {\left\langle {(A_0 - {{\,\mathrm{\text {Id}}\,}})y},{B^*y}\right\rangle }, \end{aligned} \end{aligned}$$

where we used the definition (B.20) and the equivalence $\left( B^{-1}\right) ^* = \left( B^*\right) ^{-1}$. Inserting the definition (B.22) of the operator $B^*$, we transform

B.31 $$\begin{aligned} {\left\langle {x},{A_1x}\right\rangle }= &   \Vert B^*y\Vert ^2 + \phi {\left\langle {(A_0 - {{\,\mathrm{\text {Id}}\,}})y},{B^*y}\right\rangle } \nonumber \\= &   \Vert y + (1-\phi ) P(A_0 - {{\,\mathrm{\text {Id}}\,}})y\Vert ^2 + \phi {\left\langle {(A_0 - {{\,\mathrm{\text {Id}}\,}})y},{y + (1-\phi ) P(A_0 - {{\,\mathrm{\text {Id}}\,}})y}\right\rangle }\nonumber \\= &   \Vert y\Vert ^2 + 2(1-\phi ) {\left\langle {y},{P(A_0 - {{\,\mathrm{\text {Id}}\,}})y}\right\rangle } + (1-\phi )^2 \Vert P(A_0 - {{\,\mathrm{\text {Id}}\,}})y\Vert ^2 \nonumber \\  &   \quad {+} \, \phi {\left\langle {(A_0 - {{\,\mathrm{\text {Id}}\,}})y},{y}\right\rangle } + \phi (1-\phi ) {\left\langle {(A_0 - {{\,\mathrm{\text {Id}}\,}})y},{P(A_0 - {{\,\mathrm{\text {Id}}\,}})y}\right\rangle }. \end{aligned}$$

rthogonality (3.2) of the projector *P*, the identity

B.32 $$\begin{aligned} {\left\langle {(A_0 - {{\,\mathrm{\text {Id}}\,}})y},{P(A_0 - {{\,\mathrm{\text {Id}}\,}})y}\right\rangle } = \Vert P(A_0 - {{\,\mathrm{\text {Id}}\,}})y\Vert ^2 \end{aligned}$$

holds. Moreover, we calculate

B.33 $$\begin{aligned} (1-\phi )^2 + \phi (1-\phi ) = 1 - 2\phi + \phi ^2 + \phi - \phi ^2 = 1 - \phi . \end{aligned}$$

Therefore, we continue to manipulate the equation

B.34 $$\begin{aligned} \begin{aligned} {\left\langle {x},{A_1x}\right\rangle } =&\phantom {+} (1-\phi )\left[ \Vert y\Vert ^2 + 2 {\left\langle {y},{P(A_0 - {{\,\mathrm{\text {Id}}\,}})y}\right\rangle } + \Vert P(A_0 - {{\,\mathrm{\text {Id}}\,}})y\Vert ^2\right] \\&{+}\, \phi \left[ \Vert y\Vert ^2 + {\left\langle {(A_0 - {{\,\mathrm{\text {Id}}\,}})y},{y}\right\rangle }\right] \\ =&\phantom {+} (1-\phi ) \Vert y + P(A_0 - {{\,\mathrm{\text {Id}}\,}})y\Vert ^2 + \phi \,{\left\langle {(A_0y},{y}\right\rangle }, \end{aligned} \end{aligned}$$

and arrive at the statement (B.21) which was to be shown.

### The explicit formula for a coated laminate

The purpose of this section is to establish the formula (3.12)

B.35 $$\begin{aligned} A_m = {{\,\mathrm{\text {Id}}\,}}+ \prod _{i=1}^m \phi _i \left[ {{\,\mathrm{\text {Id}}\,}}+ (A_0 - {{\,\mathrm{\text {Id}}\,}})\sum _{i=1}^m (1-\phi _i) \prod _{j=1}^{i-1} \phi _j P_i\right] ^{-1}(A_0 - {{\,\mathrm{\text {Id}}\,}}) \end{aligned}$$

as a consequence of the definition (3.11)

B.36 $$\begin{aligned} A_{m} = {{\,\mathrm{\text {Id}}\,}}+ \phi _{m} \left[ {{\,\mathrm{\text {Id}}\,}}+ (1 - \phi _{m}) (A_{m-1} - {{\,\mathrm{\text {Id}}\,}}) P_{m}\right] ^{-1} (A_{m-1} - {{\,\mathrm{\text {Id}}\,}}), \quad m=1,2,\ldots \end{aligned}$$

In this section, we use that the operator (3.13)

B.37 $$\begin{aligned} B_m = {{\,\mathrm{\text {Id}}\,}}+ (A_0 - {{\,\mathrm{\text {Id}}\,}})\sum _{i=1}^m (1-\phi _i) \prod _{j=1}^{i-1} \phi _j P_i \end{aligned}$$

is invertible, see Appendix B.5.

      We proceed via mathematical induction. For m=1, both equations (B.35) and (B.36) coincide. So, suppose that Eq. (B.36) holds for the index $m \in \mathbb {N}$, and the subsequent operator is defined via

B.38 $$\begin{aligned} A_{m+1} = {{\,\mathrm{\text {Id}}\,}}+ \phi _{m+1} \left[ {{\,\mathrm{\text {Id}}\,}}+ (1 - \phi _{m+1}) (A_{m} - {{\,\mathrm{\text {Id}}\,}}) P_{m+1}\right] ^{-1} (A_{m} - {{\,\mathrm{\text {Id}}\,}}). \end{aligned}$$

We may write Eq. (B.37) in the form

B.39 $$\begin{aligned} A_{m} - {{\,\mathrm{\text {Id}}\,}}= f_m B_m^{-1} (A_0 - {{\,\mathrm{\text {Id}}\,}}) \quad \text {with} \quad f_m = \phi _1 \phi _2 \cdots \phi _m. \end{aligned}$$

Inserting this formula into Eq. (B.38) yields

B.40 $$\begin{aligned} \begin{aligned} A_{m+1}&= {{\,\mathrm{\text {Id}}\,}}+ \phi _{m+1} \left[ {{\,\mathrm{\text {Id}}\,}}+ (1 - \phi _{m+1}) (A_{m} - {{\,\mathrm{\text {Id}}\,}}) P_{m+1}\right] ^{-1} (A_{m} - {{\,\mathrm{\text {Id}}\,}})\\&= {{\,\mathrm{\text {Id}}\,}}+ \phi _{m+1} \left[ {{\,\mathrm{\text {Id}}\,}}+ (1 - \phi _{m+1}) f_m B_m^{-1} (A_0 - {{\,\mathrm{\text {Id}}\,}}) P_{m+1}\right] ^{-1} f_m B_m^{-1} (A_0 - {{\,\mathrm{\text {Id}}\,}})\\&= {{\,\mathrm{\text {Id}}\,}}+ f_{m+1} \left[ B_m + (1 - \phi _{m+1}) f_{m+1} (A_0 - {{\,\mathrm{\text {Id}}\,}}) P_{m+1}\right] ^{-1} (A_0 - {{\,\mathrm{\text {Id}}\,}}). \end{aligned} \end{aligned}$$

For the term under the inverse, we compute

B.41 $$\begin{aligned} \begin{aligned}&\phantom {=\,} B_m + (1 - \phi _{m+1}) f_{m+1} (A_0 - {{\,\mathrm{\text {Id}}\,}}) P_{m+1}\\&{=\,} {{\,\mathrm{\text {Id}}\,}}+ (A_0 - {{\,\mathrm{\text {Id}}\,}})\sum _{i=1}^m (1-\phi _i) \prod _{j=1}^{i-1} \phi _j P_i + (1 - \phi _{m+1}) f_{m+1} (A_0 - {{\,\mathrm{\text {Id}}\,}}) P_{m+1} \\&{=\,} {{\,\mathrm{\text {Id}}\,}}+ (A_0 - {{\,\mathrm{\text {Id}}\,}})\sum _{i=1}^{m+1} (1-\phi _i) \prod _{j=1}^{i-1} \phi _j P_i\\&{=\,} B_{m+1}. \end{aligned} \end{aligned}$$

In particular, the validity of Eq. (B.35) with index m+1 in place of *m* is established.

### Invertibility of the operator in Eq. (3.13)

The purpose of this section is to establish the invertibility of the bounded linear operator (3.13)

B.42 $$\begin{aligned} B = {{\,\mathrm{\text {Id}}\,}}+ (1-f)(A_0 - {{\,\mathrm{\text {Id}}\,}})\,\sum _{i=1}^m w_i P_i, \end{aligned}$$

where f∈(0,1] refers to a volume fraction, $P_i$ denote orthogonal projectors and $w_i$ are positive coefficients which sum to unity:

B.43 $$\begin{aligned} w_i > 0 \quad (i=1,2,\ldots ,m) \quad \text {and} \quad \sum _{i=1}^m w_i = 1. \end{aligned}$$

For conciseness of notation, we introduced the operator

B.44 $$\begin{aligned} C = \sum _{i=1}^m w_i P_i \end{aligned}$$

which is self-adjoint, non-expansive, but no projector (!). We show that the kernels of both the operator *B* and its adjoint

B.45 $$\begin{aligned} B^* = {{\,\mathrm{\text {Id}}\,}}+ (1-f)C(A_0 - {{\,\mathrm{\text {Id}}\,}}) \end{aligned}$$

are trivial.

      Let *x* be an element of the kernel of the operator *B*,

B.46 $$\begin{aligned} 0 = Bx \equiv x +(1-f)A_0 C x - (1-f) C x. \end{aligned}$$

Taking the inner product with the vector *Cx*, we obtain the equation

B.47 $$\begin{aligned} 0 = {\left\langle {x},{Cx}\right\rangle } + (1-f) {\left\langle {Cx},{A_0 C x}\right\rangle } - (1-f) \Vert Cx\Vert ^2, \end{aligned}$$

which we may split in the form

B.48 $$\begin{aligned} 0 = f {\left\langle {x},{Cx}\right\rangle } + (1-f) {\left\langle {Cx},{A_0 C x}\right\rangle } + (1-f) \left( {\left\langle {x},{Cx}\right\rangle } - \Vert Cx\Vert ^2\right) , \end{aligned}$$

Suppose that the inequality

B.49 $$\begin{aligned} \Vert Cx\Vert ^2 \le {\left\langle {x},{Cx}\right\rangle } \end{aligned}$$

holds. Then, we may estimate from above

B.50 $$\begin{aligned} \begin{aligned} 0&= f {\left\langle {x},{Cx}\right\rangle } + (1-f) {\left\langle {Cx},{A_0 C x}\right\rangle } + (1-f) \left( {\left\langle {x},{Cx}\right\rangle } - \Vert Cx\Vert ^2\right) \\&\ge f {\left\langle {x},{Cx}\right\rangle }\\&\equiv f\sum _{i=1}^m w_i \, {\left\langle {x},{P_i x}\right\rangle }\\&\equiv f\sum _{i=1}^m w_i \, \Vert P_i x\Vert ^2\\ \end{aligned} \end{aligned}$$

where we used that the operator $A_0$ is positive semi-definite. As the coefficients (B.43) are positive, we must have $P_i x = 0$ for all i=1,2,…,m. In particular, the vector *x* lies in the kernel of the operator *C*

B.51 $$\begin{aligned} C x = \sum _{i=1}^m w_i \, \underbrace{P_i x}_{=0} = 0. \end{aligned}$$

In view of Eq. (B.46), the vector *x* must vanish.

Thus, it remains to establish the inequality (B.49), which we may write more explicitly in the form

B.52 $$\begin{aligned} \left\| \sum _{i=1}^m w_i \, P_i x\right\| ^2 \le \sum _{i=1}^m w_i \, {\left\langle {x},{P_ix}\right\rangle } \equiv \sum _{i=1}^m w_i \, \Vert P_ix\Vert ^2. \end{aligned}$$

      However, the latter follows directly from Jensen’s inequality [63]

B.53 $$\begin{aligned} g\left( \sum _{i=1}^m w_i \, y_i\right) \le \sum _{i=1}^m w_i g(y_i), \end{aligned}$$

applied to the convex function

B.54 $$\begin{aligned} g: H \rightarrow \mathbb {R}, \quad x \mapsto \Vert x\Vert ^2, \end{aligned}$$

for $$y_i \equiv P_i x$$.

      We turn our attention to the kernel of the operator $B^*$. Let *x* be an element of the kernel, i.e., the equation

B.55 $$\begin{aligned} 0 = B^*x \equiv x + (1-f)CA_0x - (1-f)Cx \end{aligned}$$

holds, see Eq. (B.45). Taking an inner product with the vector $A_0 x$ leads to the identity

B.56 $$\begin{aligned} 0 = {\left\langle {x},{A_0x}\right\rangle } + (1-f){\left\langle {A_0x},{CA_0x}\right\rangle } - (1-f){\left\langle {A_0x},{Cx}\right\rangle }, \end{aligned}$$

i.e., we may express

B.57 $$\begin{aligned} (1-f){\left\langle {A_0x},{Cx}\right\rangle } = {\left\langle {x},{A_0x}\right\rangle } + (1-f){\left\langle {A_0x},{CA_0x}\right\rangle }. \end{aligned}$$

As both operators $A_0$ and *C* are positive semi-definite, the left-hand side must be nonnegative, as well

B.58 $$\begin{aligned} (1-f){\left\langle {A_0x},{Cx}\right\rangle } \ge 0. \end{aligned}$$

Let us return to Eq. (B.55) and take the inner product with the vector *x*

B.59 $$\begin{aligned} 0 = \Vert x\Vert ^2 + (1-f)\underbrace{{\left\langle {x},{CA_0x}\right\rangle }}_{={\left\langle {Cx},{A_0x}\right\rangle }} - (1-f){\left\langle {x},{Cx}\right\rangle } \end{aligned}$$

By the previous finding (B.58), the middle term is nonnegative, and we may estimate

B.60 $$\begin{aligned} \begin{aligned} 0&= \Vert x\Vert ^2 + (1-f)\underbrace{{\left\langle {x},{CA_0x}\right\rangle }}_{={\left\langle {Cx},{A_0x}\right\rangle }} - (1-f){\left\langle {x},{Cx}\right\rangle }\\&\ge \Vert x\Vert ^2 - (1-f){\left\langle {x},{Cx}\right\rangle }\\&= f\Vert x\Vert ^2 + (1-f)\left[ \Vert x\Vert ^2 - {\left\langle {x},{Cx}\right\rangle }\right] \\&\ge f\Vert x\Vert ^2, \end{aligned} \end{aligned}$$

where we used the estimate

B.61 $$\begin{aligned} \begin{aligned} {\left\langle {x},{Cx}\right\rangle } = \sum _{i=1}^m w_i \, {\left\langle {x},{P_i x}\right\rangle } = \sum _{i=1}^m w_i \, \Vert P_i x\Vert ^2 \le \sum _{i=1}^m w_i \, \Vert x\Vert ^2 = \Vert x\Vert ^2, \end{aligned} \end{aligned}$$

implied by the orthogonality of the projectors $P_i$. In particular, the estimate (B.60) shows that the vector *x* vanishes, concluding the argument.

### Characterization of the kernel of the operator (3.19)

In this section, we show that any vector *x* in the kernel of the operator (3.19)

B.62 $$\begin{aligned} A = {{\,\mathrm{\text {Id}}\,}}- f \left[ {{\,\mathrm{\text {Id}}\,}}- (1-f)C\right] ^{-1} \end{aligned}$$

for f∈(0,1) satisfies the equation (3.22)

B.63 $$\begin{aligned} C x = x. \end{aligned}$$

Consider an element x∈H of the kernel of the operator *A*, i.e.,

B.64 $$\begin{aligned} 0 = Ax \equiv x - f \left[ {{\,\mathrm{\text {Id}}\,}}- (1-f)C\right] ^{-1}x. \end{aligned}$$

Thus, the vector *x* satisfies the equation

B.65 $$\begin{aligned} x = f \left[ {{\,\mathrm{\text {Id}}\,}}- (1-f)C\right] ^{-1}x, \quad \text {or, equivalently,} \quad \left[ {{\,\mathrm{\text {Id}}\,}}- (1-f)C\right] x = f \,x. \end{aligned}$$

Expanding the left-hand side of the latter equation yields

B.66 $$\begin{aligned} x - (1-f)C\,x = f \,x, \quad \text {i.e.,} \quad (1-f)x = (1-f)C\,x. \end{aligned}$$

Dividing by the prefactor 1-f yields the claim.

### Characterizing eigenvectors of the operator (3.17) for the eigenvalue 1

This section is devoted to showing the equivalence (3.23) of the two statements

B.67 $$\begin{aligned} \sum _{i=1}^m c_i \,P_i x = x \end{aligned}$$

and

B.68 $$\begin{aligned} x = P_i \, x \quad \text {for all} \quad i=1,2,\ldots ,m, \end{aligned}$$

for a vector x∈H and where $c_i$ are positive constants which sum to unity and the $P_i$ are orthogonal projectors.

Suppose that Eq. (B.68) holds. Then, we observe

B.69 $$\begin{aligned} \sum _{i=1}^m c_i \,\underbrace{P_i x}_{=x} = x\underbrace{\sum _{i=1}^m c_i}_{=1} = x, \end{aligned}$$

i.e., the condition (B.67) follows. Conversely, suppose the equation (B.67) holds and let us fix an index $i \in \{1,2,\ldots ,m\}$. By the Pythagorean Theorem (3.3) in our case,

B.70 $$\begin{aligned} \Vert x\Vert ^2 = \Vert P_i x\Vert ^2 + \Vert Q_i x\Vert ^2 \quad \text {with} \quad Q_i = {{\,\mathrm{\text {Id}}\,}}- P_i, \end{aligned}$$

on the one hand, we have the estimate

B.71 $$\begin{aligned} \Vert P_i x\Vert \le \Vert x\Vert . \end{aligned}$$

On the other hand, as we assumed the validity of the representation (B.67)

B.72 $$\begin{aligned} x = \sum _{j=1}^m c_j \,P_j x = c_i \, P_i x + \sum _{j=1, j\ne i}^m c_j \,P_j x , \end{aligned}$$

which implies the estimate

B.73 $$\begin{aligned} \Vert x \Vert \le \left\| c_i \, P_i x + \sum _{j=1, j\ne i}^m c_j \,P_j x \right\| \le c_i \, \Vert P_i x\Vert + \sum _{j=1, j\ne i}^m c_j \,\Vert P_j x \Vert \end{aligned}$$

via the triangle inequality and the positivity of the coefficients $c_j$. As the operators $P_j$ are orthogonal projectors, the estimate (B.71) implies

B.74 $$\begin{aligned} \Vert x \Vert \le c_i \, \Vert P_i x\Vert + \sum _{j=1, j\ne i}^m c_j \, \underbrace{\Vert P_j x \Vert }_{\le \Vert x\Vert } \le c_i \, \Vert P_i x\Vert + \sum _{j=1, j\ne i}^m c_j \,\Vert x \Vert . \end{aligned}$$

Furthermore, computing the sum

B.75 $$\begin{aligned} \sum _{j=1, j\ne i}^m c_j = 1 - c_i \end{aligned}$$

permits us to conclude the estimate

B.76 $$\begin{aligned} c_i \, \Vert x \Vert \le c_i \, \Vert P_i x\Vert , \quad \text {i.e.,} \quad \Vert x\Vert \le \Vert P_i x\Vert . \end{aligned}$$

Combined with the estimate (B.71), we consequently obtain the identity

B.77 $$\begin{aligned} \Vert x\Vert = \Vert P_i x\Vert , \end{aligned}$$

Inserting Eq. (B.77) into the Pythagorean identity (B.70) yields

B.78 $$\begin{aligned} \Vert Q_i x\Vert = 0 \quad \Leftrightarrow \quad Q_i x = 0. \end{aligned}$$

which implies $x = P_i x$, which was to be shown.
